# Evaluation of an App-Based Mobile Triage System for Mass Casualty Incidents: Within-Subjects Experimental Study

**DOI:** 10.2196/65728

**Published:** 2024-11-21

**Authors:** Martin Schmollinger, Jessica Gerstner, Eric Stricker, Alexander Muench, Benjamin Breckwoldt, Manuel Sigle, Peter Rosenberger, Robert Wunderlich

**Affiliations:** 1 School of Informatics Reutlingen University Reutlingen Germany; 2 University Department of Anesthesiology and Intensive Care Medicine University Hospital Tübingen Eberhard Karls University Tübingen Germany; 3 Department of Cardiology and Angiology University Hospital Tübingen Eberhard Karls University Tübingen Germany; 4 German Society for Disaster Medicine (Deutsche Gesellschaft für Katastrophenmedizin) Kirchseeon Germany

**Keywords:** disaster medicine, mass casualty incidents, digitalization, triage, Germany, mobile triage app

## Abstract

**Background:**

Digitalization in disaster medicine holds significant potential to accelerate rescue operations and ultimately save lives. Mass casualty incidents demand rapid and accurate information management to coordinate effective responses. Currently, first responders manually record triage results on patient cards, and brief information is communicated to the command post via radio communication. Although this process is widely used in practice, it involves several time-consuming and error-prone tasks. To address these issues, we designed, implemented, and evaluated an app-based mobile triage system. This system allows users to document responder details, triage categories, injury patterns, GPS locations, and other important information, which can then be transmitted automatically to the incident commanders.

**Objective:**

This study aims to design and evaluate an app-based mobile system as a triage and coordination tool for emergency and disaster medicine, comparing its effectiveness with the conventional paper-based system.

**Methods:**

A total of 38 emergency medicine personnel participated in a within-subject experimental study, completing 2 triage sessions with 30 patient cards each: one session using the app-based mobile system and the other using the paper-based tool. The accuracy of the triages and the time taken for each session were measured. Additionally, we implemented the User Experience Questionnaire along with other items to assess participants’ subjective ratings of the 2 triage tools.

**Results:**

Our 2 (triage tool) × 2 (tool order) mixed multivariate analysis of variance revealed a significant main effect for the triage tool (*P*<.001). Post hoc analyses indicated that participants were significantly faster (*P*<.001) and more accurate (*P*=.005) in assigning patients to the correct triage category when using the app-based mobile system compared with the paper-based tool. Additionally, analyses showed significantly better subjective ratings for the app-based mobile system compared with the paper-based tool, in terms of both school grading (*P*<.001) and across all 6 scales of the User Experience Questionnaire (all *P*<.001). Of the 38 participants, 36 (95%) preferred the app-based mobile system. There was no significant main effect for tool order (*P*=.24) or session order (*P*=.06) in our model.

**Conclusions:**

Our findings demonstrate that the app-based mobile system not only matches the performance of the conventional paper-based tool but may even surpass it in terms of efficiency and usability. This advancement could further enhance the potential of digitalization to optimize processes in disaster medicine, ultimately leading to the possibility of saving more lives.

## Introduction

### Background

In recent years, the number of disasters involving mass casualty incidents (MCIs) has significantly increased [1], driven by factors such as terrorism, climate change, and war. While disaster medicine procedures are well-established and effective, there remains potential for optimization through digitalization [2]. Currently, information collected manually and on paper during an MCI is often incomplete, sometimes disorganized, and potentially unreliable. This includes critical details such as the number of patients, their locations, identifying characteristics, and injury severity. These limitations can hinder decision makers’ ability to obtain timely and accurate information essential for an effective response. This information, especially from rescue teams who are first on the scene, is crucial for effective mass casualty and disaster management, as it can save lives and provide valuable insights for scientific research to enhance future operations [3]. First responders must prioritize patients based on injury severity to ensure that those in critical condition receive immediate attention. Incident commanders coordinate rescue resources and communicate with hospitals about capacity, requiring a comprehensive overview of patient numbers, injury severity, and locations. Upon arrival at the incident scene, rescue teams search for casualties and categorize patients [4] using triage algorithms such as Simple Triage and Rapid Treatment (START) [5], modified Simple Triage and Rapid Treatment (mSTART) [6], and jumpSTART for children [7,8], classifying them by health status with color codes (Textbox 1).

**Triage categories following the modified Simple Triage and Rapid Treatment algorithm.**

1. Red
  - Patients with life-threatening injuries that immediately need an adequate treatment.
2. Yellow
  - Patients that are seriously injured and not able to walk. The treatment is still urgent but can be delayed after the first group.
3. Green
  - Patients that are slightly injured, able to walk, and can be treated later. Sometimes they are also called “walking wounded.”
4. Black
  - Deceased casualties or casualties that are dying and only need supportive treatment.
5. BLUE
  - Sometimes blue is used for patients that have no chance of surviving under the given circumstances considering the available resources.

Biographic data and initial treatments are recorded on handwritten cards attached to patients, aiding in communication and tracking. All information is relayed to incident commanders via radio, who coordinate with various rescue organizations and manage resources and patient transport based on the triage data [9,10]. Rescue services establish areas for admission, treatment, decontamination, and transportation, where patients are re-triaged to monitor changes in their health status [11]. This process ensures that the most critical patients receive timely and appropriate care.

### Weaknesses and Problems With the Current Emergency Medical Care During MCIs

Triage data collected by rescue teams are currently communicated to the command post via radio, which must be concise and minimal due to time constraints [12]. Radio communication is not suitable for transmitting large volumes of data. Communication from rescue teams to incident commanders and dispatch centers follows a many-to-one pattern, requiring recipients to manually compile information—a process that is both time-consuming and prone to error. During triage, rescue teams gather additional data, including patient biographic information and minor treatment details. Transmitting all this information via radio is too time-consuming, so only the most critical data are communicated, resulting in incomplete information that can hinder effective disaster management [13,14]. Incident commanders often need to contact rescue teams for missing details, adding to communication time. Furthermore, verbal descriptions of patient locations are less precise than GPS tracking, complicating rapid evacuations. Rescue teams move quickly from patient to patient and can only intermittently update the command post on their findings, causing delays in situational awareness. This slow development of an overall view of the disaster scene can lead to outdated or incorrect decisions, requiring reorganization and resulting in time loss [15]. The manual, communication-intensive processes reduce work quality and increase task execution times, which can dramatically impact patient outcomes. Automating the process with a software system can help address these issues by providing a common, centralized data source accessible to all involved parties [16].

### The App-Based Mobile System “KatApp”

The weaknesses in the current rescue process stem from the absence of a common, centralized data source accessible to all involved parties. If rescue teams utilized software for triaging patients, the results could be immediately stored in a shared data source. Incident commanders connected to this data source would have access to real-time information tailored to their needs, thereby eliminating several weaknesses through process automation. This would eliminate the need for manual compilation of information and facilitate the creation of complete data sets, minimizing data incompleteness and reducing additional communication costs. The software could also automatically track GPS coordinates of triage locations and present real-time data, enhancing the overall quality and speed of the process. By removing several manual tasks, the overall quality of work would improve, reducing errors and allowing more time for critical tasks, thereby accelerating process execution. From a functional perspective, developing such a software system may seem straightforward; however, several nonfunctional requirements must also be considered, including usability, data security, data privacy, and system availability. The software must be intuitive and easy to use under stressful conditions, such as low visibility (eg, bright sunlight or darkness). Additionally, data security and privacy safeguards must be in place to prevent unauthorized access to sensitive patient information, and the system must be reliable and instantly accessible in real-time disaster scenarios.

These requirements are essential for the system to be effective in real disaster scenarios.

**Figure 1:**
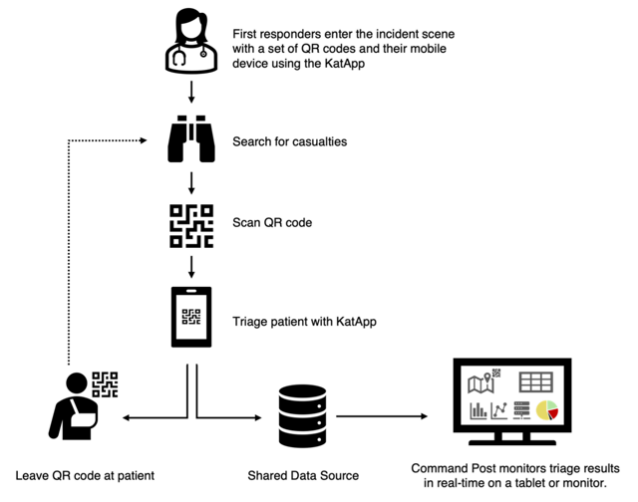
Digital triage procedure using the KatApp Software System.

To address these requirements, we utilized software prototyping [17] to design and develop a prototype named “KatApp.” This prototype was tested in several disaster medicine training sessions, during which we collected both quantitative data and qualitative feedback for evaluation. The system uses smartphones for triage and tablets for the command post, all connected to a shared cloud-based architecture. Applications running on the client side are part of the front-end layer and utilize services from the backend layer to read or write data, with the backend services relying on a persistence layer for permanent data storage. To connect patients with their digital data records during a disaster, rescue teams scan a patient-specific QR code before starting triage, seamlessly linking patients to their digital records (Figure 1).

The software architecture of the KatApp system follows a modern cloud-based architecture style, integrating backend and front-end components. The front-end layer comprises 2 client applications: a native app for triaging patients, used by rescue teams, and a web application that implements a dashboard for the command post. Both client applications access services from the backend layer, with the native app using a REST (Representational State Transfer) application programming interface and the web application connecting via a web socket connection.

The triage app is developed using Google’s Flutter framework [18], which supports multiple platforms from a single codebase—an essential feature for training and future use by different organizations. The app’s user interface is designed for ease of use during triage, transitioning from flowchart-based designs to a page flow that represents the triage algorithms. In addition to implementing the triage algorithms (mSTART and jumpSTART), the app includes several features such as day and night modes; using the smartphone flashlight to easily scan the QR code, even at night; and an overview of completed triages. The command post dashboard was developed as a web application using the React framework [19,20]. It displays information on the exact GPS locations of patients using OpenStreetMap [21], along with a list of patients who have been triaged and the time elapsed since the start of triage handling, together with the current time.

The dashboard application connects to the backend service using a web socket connection. As soon as new triage records are received by the backend service from the rescue teams’ apps, they are pushed to the dashboard application through this connection. This ensures that the command post always has the most current information without the need to manually pull data. The system’s design is well-suited for serverless computing [22,23], as it is only required during real disasters or training sessions. This allows for billing based solely on active usage, which aligns with the sporadic nature of MCIs and has the potential to reduce overall operational expenses.

After prototyping KatApp, several functionality tests were conducted under realistic conditions, including the use of waterproof devices and gloves. These tests were followed by training sessions aimed at gathering qualitative feedback to refine the prototype.

Logging functionalities were subsequently added to measure triage time and accuracy. Early usability insights were incorporated, and the system was standardized for evaluation in a within-subjects experimental study comparing it with the paper-based triage method. Table 1 presents the early implementations of our prototype in pilot studies. Several usability insights for the app and dashboard were promptly added to the backlog. To standardize the evaluation of our refined prototype against the conventional paper-based triage tool, we conducted an experiment using a within-subjects design.

Based on the insights gained from these early implementations, we made several improvements to the KatApp prototype. These refinements included enhancements to the user interface, optimization of the triage algorithms, and improvements to the data logging capabilities. The resulting version of the app, which incorporated feedback from all 3 training sessions, was used in the subsequent comparative experiment.

**Table 1:** Early-stage implementations of the KatApp prototype.

Training session	Objective	Sample	Procedure	Results
Training 1	Obtain first qualitative user feedback	N=15 rescue service personnel, emergency physicians, and medical students	Participants triaged 60 patient cards at different locations (ie, at the location of the incident, at the treatment area, and before the release of the patient)	150 triages were made More than 90% (11/12) of the app users and 100% (4/4) of the dashboard users stated that they would like to use such a system in a real disaster
Training 2	Use a prototype in an evacuation training (earthquake) and finish the evacuation within 90 minutes	N=50 medical students as first responder teams, supervised by experienced emergency physicians	Participants triaged 18 patients (played by actors)	48 triages were made The scenario could be finished in time 2 patients were triaged in the wrong category Average time of a triage: 42 seconds
Training 3	Use prototype in an evacuation training (flood warning + gas explosion) and finish the evacuation within 75 minutes	N=50 rescue service personnel and emergency physicians	Participants triaged 23 patients (played by actors)	50 triages were made All patients were triaged in the correct category Average time of a triage: 37 seconds

### Research Objective

The study aimed to evaluate the KatApp as a triage tool for emergency and disaster medicine, which was previously designed and developed by our research group, as described in the “Background” section. The objective was to determine whether the KatApp is superior to paper-based triage tools in terms of speed, accuracy, and user experience among emergency medicine experts. We hypothesized that the KatApp would be at least as accurate as the paper-based tool, while also being faster and more user-friendly.

## Methods

### Ethical Considerations

The Ethics Committee of the Faculty of Medicine at Tübingen University Hospital approved the study (approval number 260/2024BO2). All methods were implemented in accordance with the Declaration of Helsinki, and written informed consent was obtained from all participants.

### Study Design and Setting

The study was designed as a within-subjects experimental design, with the triage tool serving as a within-subjects factor (comparing the paper-based tool and KatApp). The order of the triage tools was counterbalanced to mitigate carryover effects.

The study was conducted at the German Red Cross in Kirchentellinsfurt on behalf of the University Hospital Tübingen, in collaboration with Reutlingen University, in June 2024. Participation in the study was voluntary.

### Recruitment

Invitations to the triage training course and calls for participation in the study were sent to a mailing list from the Tübingen District Association of the German Red Cross, which includes approximately 500 email addresses of rescue service personnel, physicians, and medical students with German Red Cross Emergency Medical Technician (EMT) qualifications.

To determine the sample size for the study, a statistical power analysis was conducted using G*Power (version 3.1 for MacOS; Universität Düsseldorf) [24]. The analysis indicated a required sample size of 45 participants, with an effect size of Cohen *d*=0.5, a statistical power of 0.95, and a significance level (*P* value) of .05. It is important to note that, due to the limited number of similar publications, the statistical power analysis was based on general considerations regarding the trade-offs between the ability to detect medium-sized effects and the feasibility of obtaining a sufficiently large sample. To account for potential no-shows and dropouts, we decided to recruit an additional 10 participants, bringing the total to 55.

### Study Procedures

After participants registered for the triage training course and study, they received instructions on how to install the app and details about the study procedure 1 week before the course.

**Figure 2:**
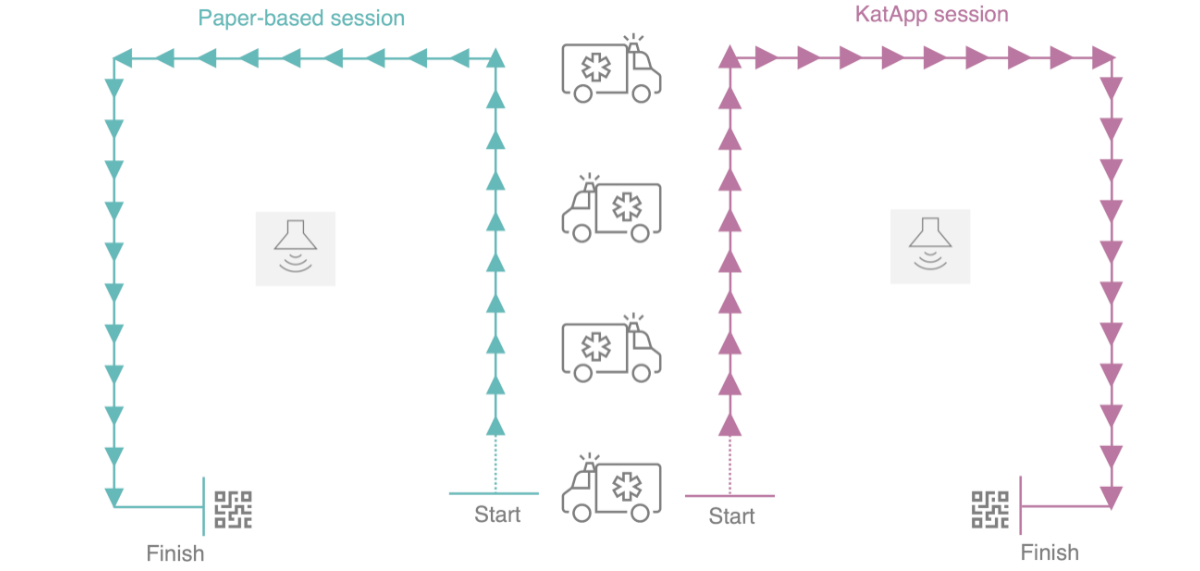
Sketch of the experimental setup. Each session consisted of 30 patient cards (marked as triangles in this sketch) that were distributed along a course of 96.32 m. Emergency vehicles and a fog machine blocked the view between the 2 courses. In addition, simulated sounds of a mass casualty incident were played via connected sound boxes that were placed in the middle of each course. Immediately after the sessions, the participants were asked to scan a QR code to complete the follow-up questionnaire.

To familiarize participants with the mSTART algorithm using both the paper-based tool and the KatApp, an introduction was provided at the beginning of the course. Participants were then asked to complete 2 triage sessions set in the context of a simulated MCI, involving a terrorist driving into the fan zone of a public screening of the UEFA (Union of European Football Associations) European Football Championship 2024. In one session, participants used the KatApp, while in the other session, they used the conventional paper-based tool. The order of the tools was randomized. Each session involved classifying 30 patient cards into 1 of 4 triage categories, with the cards distributed along a course of exactly 96.32 m for each triage tool. The 2 sessions were conducted simultaneously, with participants starting their sessions every 5 minutes. Refer to Figure 2 for a sketch of the experimental setup, Multimedia Appendix 1 for a drone photograph of the setting, and Multimedia Appendix 2 for a drone video.

After each session, participants completed a web-based questionnaire using SurveyMonkey (SurveyMonkey Inc.). Following a recovery period of approximately 30 minutes, they began the second session with the alternate triage tool.

### Patient Cards

The patient cards were initially developed using a preexisting artificial patient database [25,26] and subsequently adapted by the study team. Each card featured a pictogram to illustrate the injury pattern, along with details on injury severity (Abbreviated Injury Scale) and vital parameters, including the Glasgow Coma Scale, respiratory rate, systolic blood pressure, radial pulse, capillary refill time, walking ability, and age. Additionally, a short storyboard provided further context regarding the circumstances of the accident (Multimedia Appendix 3). To ensure comparability between the 2 sessions, the number of patient cards in each triage category was kept identical: red (6/30, 20%), yellow (13/30, 43%), green (9/30, 30%), and black (2/30, 7%). This categorization was based on empirical data from MCIs [27]. Additionally, we paired patient cards to match them in terms of overall injury severity (Abbreviated Injury Scale) and injury pattern, minimizing the impact of any differences in the patient cards on the outcome variables between the triage tools. The order of patient cards in each course was randomized using the online tool RANDOM.ORG (Randomness and Integrity Services Limited), ensuring that the randomization was consistent for each participant.

### Triage Tools

#### Paper-Based Tool

When using the conventional paper-based tool, participants were required to complete a casualty card (see Multimedia Appendix 4) from their equipment. This process involved manually indicating the triage category by folding the card and attaching it to the patient card. Additionally, participants needed to record the triage category on a documentation sheet. To assist with the triage process, they were provided with an mSTART checklist [6].

#### KatApp

When using the KatApp, participants were required to attach a QR code from their equipment to each patient. They then scanned the QR code with the KatApp’s QR code scanner and conducted the triage process as previously described (see Figure 1 and Multimedia Appendix 5). Participants used their smartphones, which were connected to the mobile network, allowing the triage results to be immediately forwarded to a central dashboard.

#### System Architecture Behind the KatApp

**Figure 3:**
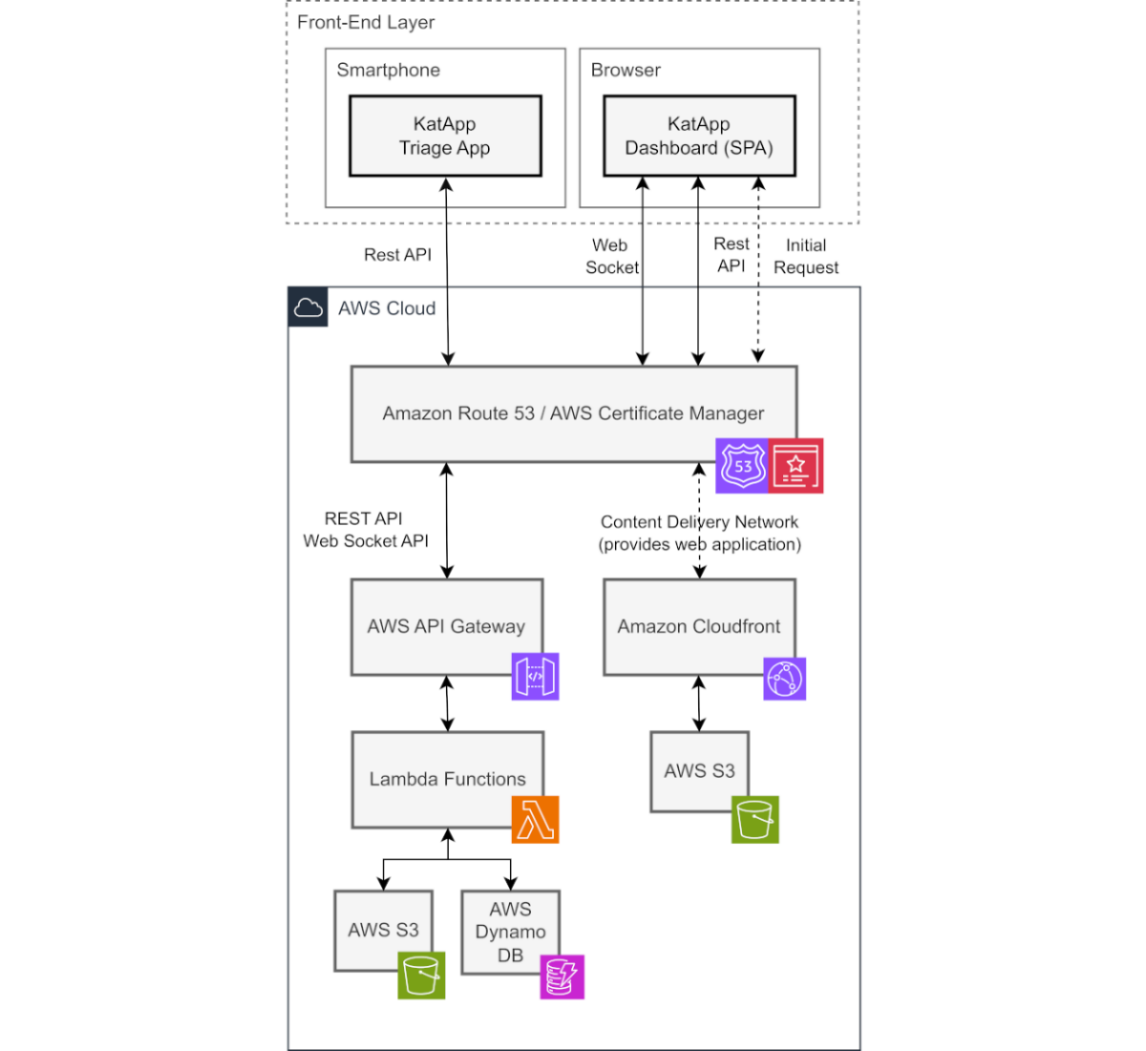
Schematic overview of the KatApp architecture without deployment and identity and access management. API: application programming interface; AWS: Amazon Web Services; DB: database; SPA: single-page application.

All software components are managed using repositories on GitHub (GitHub, Inc.). Automatic deployments are facilitated by GitHub Actions and custom scripts [28]. The iOS app is deployed to TestFlight (Apple Inc.), while the Android app is deployed to Firebase (Google LLC/Alphabet Inc.). Rescue teams can install the desired version from these platforms before training. The dashboard web application and the backend are deployed on Amazon Web Services (AWS; Amazon Web Services, Inc.). The dashboard functions as a web application hosted on AWS, while the backend services are implemented as AWS Lambda functions [29]. These services utilize AWS DynamoDB for user management, logging, and triage records, and AWS S3 for binary data storage. Figure 3 provides a detailed depiction of the architecture.

The KatApp system operates as a distributed system, dependent on the connectivity of its various nodes, which include the mobile devices used by rescue teams, the tablets at the command post, and the backend services. In standard operation mode, it is assumed that an internet connection is accessible at the incident scene, enabling the mobile apps used by rescue teams to access the web application and backend services hosted in the cloud. The dashboard web application is accessible through browsers on tablets or computers at the command post, allowing it to establish a web socket connection to the KatApp backend. In an ideal scenario, this operational mode would suffice. However, as highlighted by the fallacies of distributed computing [30], networks can often be unreliable, particularly during disasters, when essential communication infrastructure may be compromised or overwhelmed, or in areas with inadequate network coverage. To tackle these challenges, the KatApp system incorporates a caching mechanism within the triage app, enabling rescue teams to continue triaging patients even in the absence of an internet connection. Triage records are stored locally and are automatically transmitted once connectivity is restored. Furthermore, the KatApp system features an operational mode that functions independently of an internet connection, ensuring that critical triage processes can continue uninterrupted during emergencies. A local wireless network can be established by the command post or a technical organization on-site, featuring a server that hosts the dashboard application and backend services. Mobile devices can connect to this local network, enabling the system to operate as intended. As the backend services were developed as AWS Lambda functions, they cannot be executed locally. To address this limitation, an additional abstraction layer was implemented, allowing the use of alternative open-source or custom-built components for each AWS service. This ensures that the KatApp system remains functional and efficient, even in environments where internet connectivity is compromised.

### Measuring and Outcomes

#### Duration and Quality of Triages

The study staff recorded the time at the beginning and end of each course as participants crossed the start and finish lines. This timing encompassed the entire triage process, including walking between patients, applying QR codes or attaching paper triage cards (depending on the method used), and conducting the triage assessment itself. Additionally, the number of correct triages per session was evaluated by analyzing participants’ documentation sheets from the paper-based tool, as well as the data collected by the KatApp.

#### Subjective Ratings

Participants were required to rate both triage tools using the German grading system, where a score of 1 represents “excellent” and a score of 6 indicates “insufficient.” They were also asked to express their preference between the 2 tools through a forced-choice item.

#### User Experience Questionnaire

**Figure 4:**
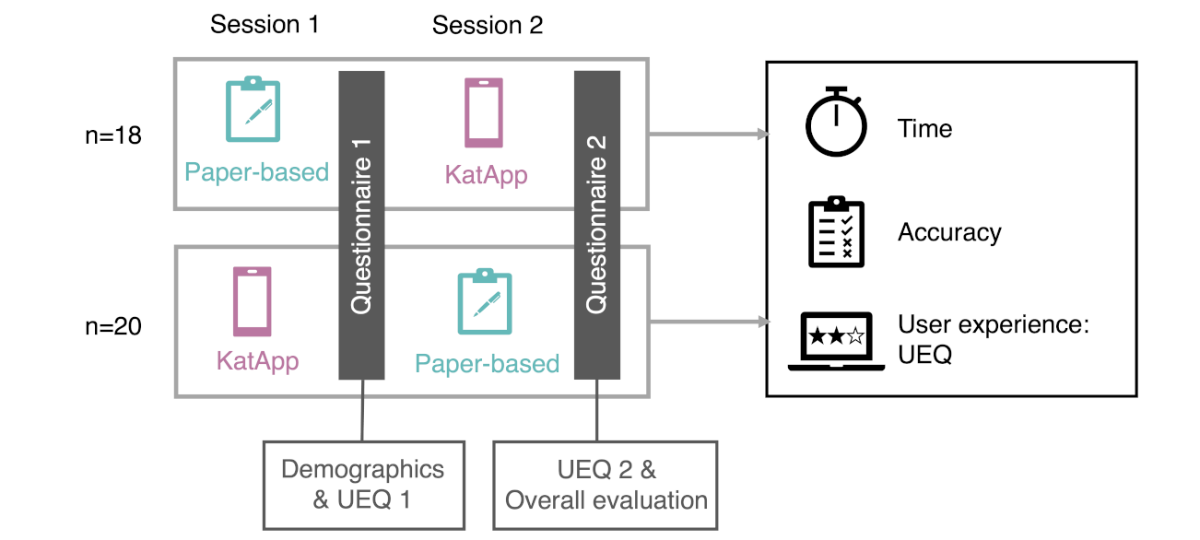
Within-subjects experimental design that was used for the evaluation of the KatApp compared with the conventional paper-based tool. Each participant conducted 2 sessions in which they had to triage 30 patient cards. The tool order was counterbalanced to compensate for possible carry-over effects. UEQ: User Experience Questionnaire.

We used the German version of the User Experience Questionnaire (UEQ) [31] to evaluate participants’ experiences with each triage tool. The UEQ comprises 26 items organized into 6 scales: Attractiveness, Perspicuity, Efficiency, Dependability, Stimulation, and Novelty. Each item is presented as a semantic differential, requiring participants to rate their responses on a 7-point Likert scale, which is coded from –3 to +3 (Multimedia Appendix 6). *Attractiveness* refers to the overall impression of the product (Does a user like or dislike the product?), whereas *Perspicuity* assesses the ease with which users can get familiar with the product and learn how to use it. *Efficiency* indicates whether users can complete their tasks without unnecessary effort, while *Dependability* refers to the sense of control, security, and predictability in the interaction. *Stimulation* measures how exciting and motivating the product is to use it, while *Novelty* assesses whether the product is creative and captures the interest of users. Figure 4 illustrates the overall experimental design.

### Statistical Analysis

SPSS version 29 for MacOS (IBM Corp.) was utilized for data management and statistical analyses.

To analyze the differences between the conventional paper-based tool and the KatApp, we performed a mixed multivariate analysis of variance (MANOVA) with the triage tool as a within-subjects factor (paper-based tool vs KatApp). To account for potential interactions between the triage tool and tool order, we included tool order (KatApp used in the first session vs the second session) as a between-subjects factor. As dependent variables, we included the overall time taken to complete each triage session, the number of correct triages, the participants’ subjective ratings of the triage tools using the German school grading system, and the results from the 6 scales of the UEQ.

In a separate repeated-measures MANOVA, we utilized the measurement time point (first vs second session) as a within-subjects factor for the same 9 dependent variables to evaluate differences between the 2 time points.

We reported the Pillai trace as the *F*-statistic in our models and conducted post hoc univariate ANOVAs for each dependent variable.

Despite some outcome variables violating the assumptions of normal distribution, we calculated MANOVAs, as this method is relatively robust against such violations to conduct MANOVAs instead of multiple ANOVAs because MANOVAs account for the complex relationships between dependent variables, offer greater statistical power, and help mitigate the issue of alpha error accumulation compared with multiple testing.

As an exploratory analysis, we calculated nonparametric correlations (Kendall tau) between age and the outcome variables to evaluate the suitability of including age as a covariate in our model. This assessment aimed to investigate the potential influences of age on the use of the app, as younger generations may be more accustomed to using smartphones and apps compared with older individuals. However, no statistically significant correlations were found (see Multimedia Appendix 7). Additionally, we chose not to include other demographic data, such as gender or profession, in our model, as they did not provide additional value concerning our study objectives and could further decrease statistical power, as previously discussed.

We established the significance level at α=.05 for our analyses.

We created raincloud plots in R (R Foundation) using the raincloudplots package [32]. This approach visualizes differences between the 2 triage tools, incorporating raw data, probability density, and key summary statistics in a transparent format.

The study was conducted in accordance with the Template for Intervention Description and Replication (TIDieR) checklist and guideline recommendations. The completed checklist is available as Multimedia Appendix 8.

## Results

### Sample Size and Exclusions

In total, 55 individuals initially registered for the course, resulting in 48 participants who attended, which reflects a no-show rate of 13% (7/55). Among those who attended, 6 participants were excluded from the statistical analyses due to incomplete triage documentation (n=3 with the paper-based tool, n=2 with the KatApp, and n=1 with both tools). Additionally, 2 participants did not complete questionnaire 1 and 2 did not complete questionnaire 2. Consequently, the final sample size for further statistical analyses comprised 38 participants.

### Sample Characteristics

Demographic characteristics of the sample are presented in Table 2. The average age of the participants was 32.5 (SD 9.2) years. Of the total 38 participants, 24 (63%) were male and 15 (39%) reported having previous experience with triaging. In terms of occupation, 14 (37%) were physicians, 17 (45%) worked as rescue service personnel, 4 (11%) were medical students with German Red Cross EMT qualifications, and 3 (8%) belonged to other groups (eg, standby service or honorary positions).

**Table 2:** Demographic characteristics of the sample.

Characteristics			Value
Age (years), mean (SD)			32.5 (9.2)
**Gender, n/N (%)**			
	Female	14/38 (37)	
	Male	24/38 (63)	
**Previous experience, n/N (%)**			
	Yes	15/38 (39)	
	No	23/38 (61)	
**Occupation, n/N (%)**			
	Physician	14/38 (37)	
	Rescue service personnel	17/38 (45)	
	Medical students with EMT^a^ qualifications	4/38 (11)	
	Other	3/38 (8)	
**Specialization (physicians**)**, n/N(%)**			
	Anesthesiology	10/14 (71)	
	Surgery	2/14 (14)	
	Neurology	1/14 (7)	
	Pediatrics	1/14 (7)	
**Additional qualification in emergency medicine (physicians), n/N (%)**			
	Yes	11/14 (79)	
	No	3/14 (21)	
**Highest qualification (rescue service personnel), n/N (%)**			
	EMT-basic	12/17 (71)	
	EMT-intermediate	1/17 (6)	
	Paramedic	4/17 (24)	

^a^EMT: Emergency Medical Technician.

### Evaluation Results

#### Overall

Findings from the 2 (triage tool) × 2 (tool order) mixed MANOVA revealed a significant main effect for the within-subjects factor of the triage tool (*F*_9,28_=70.585, *P*<.001, η^2^_p_=.958). Additionally, there was a significant interaction between the triage tool and the tool order (*F*_9,28_=4.432, *P*=.001, η^2^_p_=.588); however, no significant main effect was found for the between-subjects factor of tool order (*F*_9,28_=1.382, *P*=.24, η^2^_p_=.308).

#### Duration

**Figure 5:**
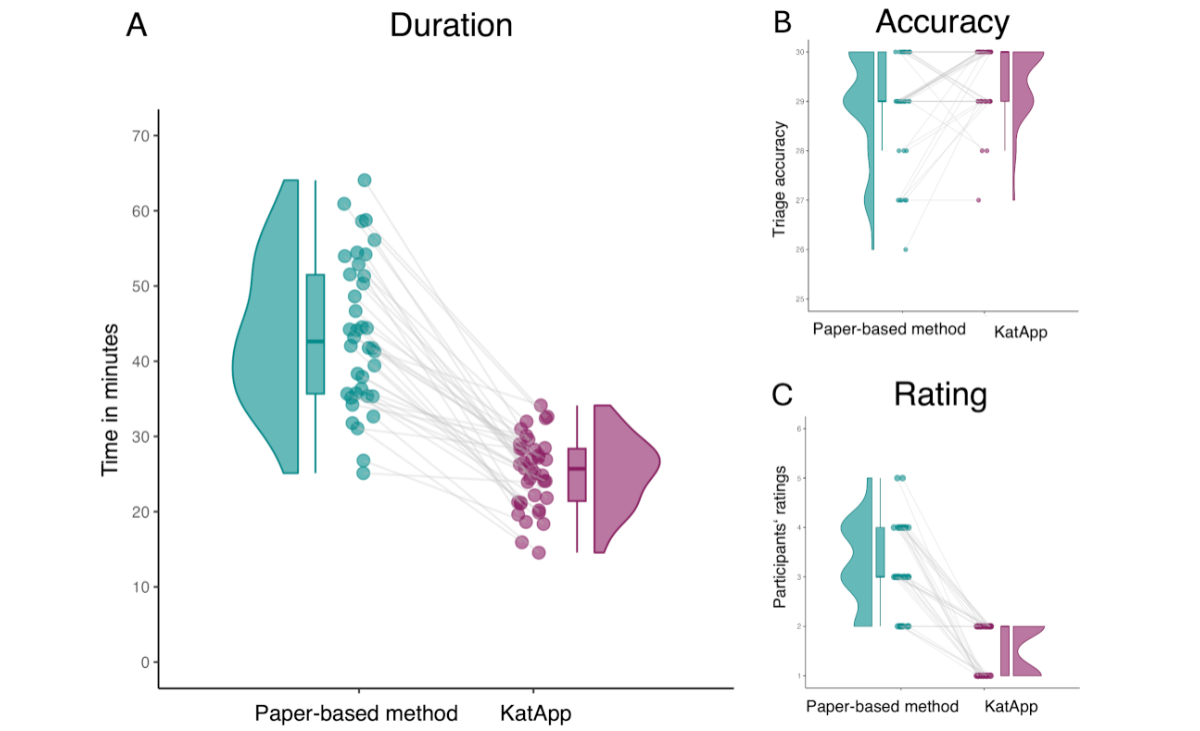
Results of the User Experience Questionnaire (UEQ) comparing the conventional paper-based tool with the KatApp regarding the 6 scales (A) Attractiveness, (B) Perspicuity, (C) Efficiency, (D) Dependability, (E) Stimulation, and (F) Novelty.

The results of the post hoc univariate ANOVA indicated that participants were significantly faster when using the KatApp compared with the paper-based tool (*F*_1,36_=229.769, *P*<.001, η^2^_p_=.865). The mean difference is illustrated in Figure 5A. In addition, a significant interaction was found between the triage tool and the tool order (*F*_1,36_=15.052, *P*<.001, η^2^_p_=.295), suggesting that the time taken for each tool was lower in the second session, although the time was consistently less for the KatApp.

#### Accuracy

The post hoc univariate ANOVA also demonstrated that participants assigned a significantly greater number of patient cards to the correct category when using the KatApp compared with the paper-based tool (*F*_1,36_=8.979, *P*=.005, η^2^_p_=.2), as illustrated in Figure 5B. Additionally, a significant interaction was observed between the triage tool and the tool order concerning triage quality (*F*_1,36_=4.731, *P*=.04, η^2^_p_=.116). This interaction indicates that the number of correct triages was higher in the second session for both tools, with the KatApp consistently yielding a greater number of correct triages.

#### Subjective Ratings

When participants rated the triage tools using the German school grading system, the post hoc univariate ANOVA revealed significantly better ratings for the KatApp (rated as excellent) compared with the paper-based tool (rated as satisfactory; *F*_1,36_=116.281, *P*<.001, η^2^_p_=.764). There was no significant interaction between the triage tool and the tool order (*F*_1,36_=0.007, *P*=.93, η^2^_p_=.000). The mean difference is illustrated in Figure 5C.

#### User Experience Questionnaire

**Figure 6:**
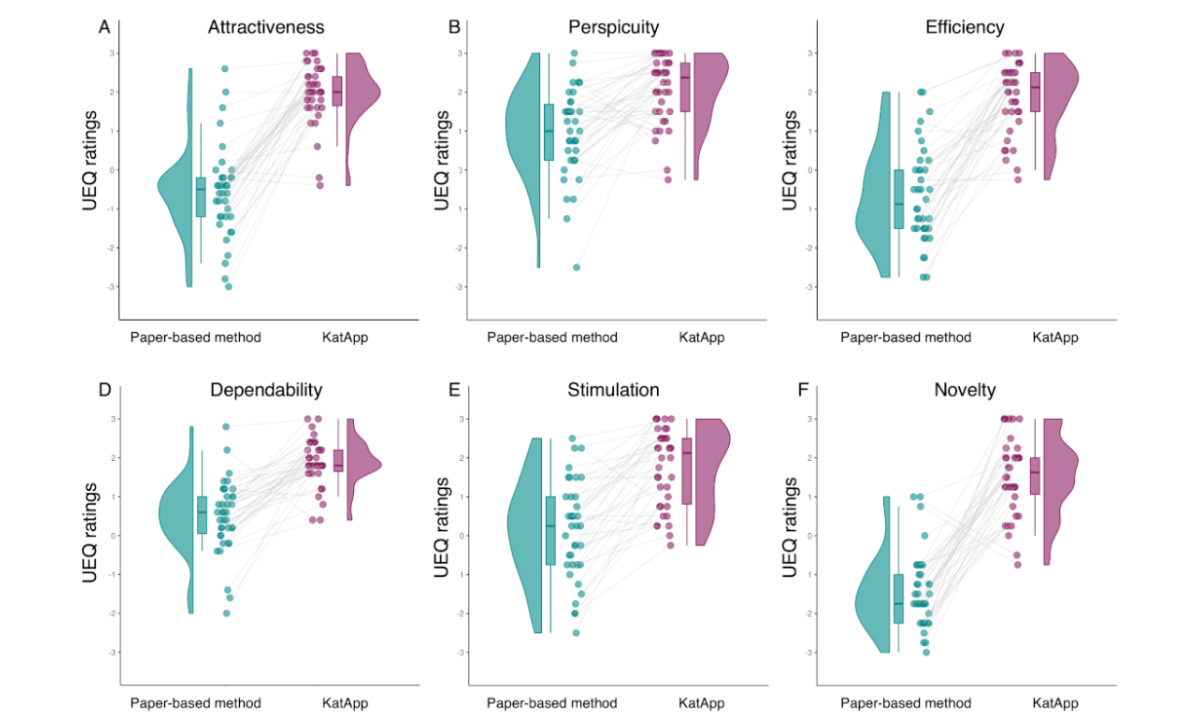
Comparison of the conventional paper-based tool with the KatApp regarding (A) duration to complete each session in minutes, (B) triage accuracy as number of correct triages out of 30, and (C) the participants’ subjective ratings as German school grades from 1 (excellent) to 6 (insufficient). UEQ: User Experience Questionnaire.

Regarding the 6 scales of the UEQ, post hoc univariate ANOVAs indicated significantly better ratings for the KatApp compared with the paper-based tool on each scale (Attractiveness: *F*_1,36_=128.812, *P*<.001, η^2^_p_=.782; Efficiency: *F*_1,36_=105.542, *P*<.001, η^2^_p_=.744; Perspicuity: *F*_1,36_=26.390, *P*<.001, η^2^_p_=.423; Dependability: *F*_1,36_=61.693, *P*<.001, η^2^_p_=.631; Stimulation: *F*_1,36_=58.422, *P*<.001, η^2^_p_=.619; and Novelty: *F*_1,36_=132.687, *P*<.001, η^2^_p_=.787). There was no significant interaction between the triage tool and the tool order for any of the UEQ scales (Attractiveness: *F*_1,36_=0.214, *P=*.65, η^2^_p_=.006; Efficiency: *F*_1,36_=0.006, *P*=.94, η^2^_p_=.000; Perspicuity: *F*_1,36_=0.636, *P*=.43, η^2^_p_=.017; Dependability: *F*_1,36_=1.762, *P*=.19, η^2^_p_=.047; Stimulation: *F*_1,36_=0.434, *P*=.51, η^2^_p_=.012; Novelty: *F*_1,36_=2.361, *P*=.13, η^2^_p_=.062). Figure 6 illustrates the differences between the tools regarding the 6 scales of the UEQ.

The mean values and corresponding statistics for the outcome measures between the 2 triage tools are presented in Table 3 and Multimedia Appendix 9 (also see [33]).

**Table 3:** Post hoc univariate analysis of variance comparison between the paper-based tool and the KatApp (N=38).

Outcome variable			Annotations in the paper-based tool, mean (SD)		Annotations in the KatApp, mean (SD)		*F* test (*df*)		*P* value		η^2^_p_
Duration (minutes)			43.71 (9.816)		25.22 (4.737)		229.769 (1, 36)		<.001		0.865
Triage accuracy			28.92 (1.099)		29.45 (0.724)		8.979 (1, 36)		.005		0.200
Subjective rating			3.26 (0.860)		1.53 (0.506)		116.281 (1, 36)		<.001		0.764
**User Experience Questionnaire**											
	Attractiveness	–0.58 (1.175)		1.96 (0.764)		128.812 (1, 36)		<.001		.782	
	Efficiency	–0.65 (1.224)		1.86 (0.900)		104.542 (1, 36)		<.001		.744	
	Perspicuity	0.94 (1.146)		2.09 (0.861)		26.390 (1, 36)		<.001		.423	
	Dependability	0.49 (0.937)		1.88 (0.600)		61.693 (1, 36)		<.001		.631	
	Stimulation	0.14 (1.251)		1.76 (0.997)		58.422 (1, 36)		<.001		.619	
	Novelty	–1.45 (0.964)		1.54 (0.999)		132.687 (1, 36)		<.001		.787	

#### Preference

When participants were asked to express their preference using a forced-choice item, an overwhelming majority (36/38, 95%) favored the KatApp, while the remaining 2 (5%) preferred the paper-based tool.

### Controlling for Order Effects

The results of the 2 (triage tool) × 2 (tool order) mixed MANOVA indicated no significant main effect for the between-subjects factor of tool order (*F*_9,28_=1.382, *P*=.24, η^2^_p_=.308). However, univariate ANOVAs for the between-subjects effect revealed no significant differences across the measures, except for the UEQ scale novelty (*F*_1,36_=6.625, *P*=.01, η^2^_p_=.155). This finding suggests that when the KatApp was used in the second session, novelty ratings were significantly higher than when it was used first.

Additionally, the repeated measures MANOVA for outcome measures at each time point, with the session as a within-subjects factor, did not show a significant main effect for session (*F*_9,29_=2.094, *P*=.06, η^2^_p_=.394). None of the post hoc analyses indicated significant differences between the first and second sessions for any of the outcome measures (Table 4).

**Table 4:** Post hoc univariate multivariate analysis of variance comparison between the results of the first and second sessions.

Compared variables			*F* test (*df*)		*P* value		η^2^_p_
Triage quality			3.357 (1, 37)		.07		.083
Time duration			3.357 (1, 37)		.26		.035
Subjective rating			0.104 (1, 37)		.75		.003
**User Experience Questionnaire**							
	Attractiveness	0.253 (1, 37)		.62		.007	
	Efficiency	0.101 (1, 37)		.75		.003	
	Perspicuity	0.165 (1, 37)		.69		.004	
	Dependability	1.151 (1, 37)		.29		.030	
	Stimulation	0.443 (1, 37)		.51		.012	
	Novelty	0.190 (1, 37)		.66		.005	

## Discussion

### Principal Findings

In this paper, we present an innovative approach to triage that replaces traditional paper-based classification with a mobile app–based system for categorizing patients in the context of MCIs. After prototyping the mobile app, we tested it in several training sessions and refined its features. The final prototype was then evaluated against the conventional paper-based tool regarding time duration, triage quality, and subjective ratings, such as user experience, using a within-subjects experimental study. Our results suggest that using this software system enables triage to be completed more quickly, easily, and accurately compared with the conventional paper-based tool. This approach addresses the time-consuming and error-prone issues associated with current procedures in disaster medicine.

The most significant and noticeable difference was the time participants required to complete the 2 triage sessions, each consisting of 30 patient cards. Using the KatApp, participants were, on average, over 18 minutes faster than when using the paper-based tool. Additionally, because command posts receive information in real time, critical decisions can be made sooner, allowing for faster patient treatment, which could ultimately save more lives. It is important to note that participants did not have to transmit their information via radio communication to the command post during the paper-based session, which would have added even more time. The requirement to fill out casualty cards manually and complete the documentation sheet likely contributed to this time difference.

Participants also found the KatApp to be more user-friendly than the conventional paper-based tool. In each of the 6 scales of the UEQ, participants rated the KatApp as “excellent” or at least “good” according to the corresponding benchmarks [33]. By contrast, the paper-based tool received ratings of “bad” or “below average.” This trend is reflected in the school grades awarded by participants: the KatApp was perceived as “excellent,” while the paper-based tool averaged a “satisfactory” grade. These results suggest that, in addition to the time advantage, the KatApp was perceived as easier to use than the paper-based tool. Furthermore, the significance of digitizing disaster medicine processes is underscored by the fact that 36 out of 38 (95%) participants indicated a preference for the KatApp over the traditional triage tool.

Surprisingly, there was also a statistically significant difference in triage accuracy between the 2 tools, indicating that participants triaged more patients correctly when using the KatApp compared with the paper-based tool. However, because the effect size for this difference was smaller than that of other measures, this result should be interpreted with caution. It is possible that the patient cards varied slightly, even though they were matched in terms of injury severity, injury pattern, and triage category. Additionally, the number of correctly assigned patient cards was high for both tools. However, some participants particularly valued the support provided by the app during triage, which may have contributed to fewer errors due to the automated application of the mSTART algorithm.

The results of our within-subjects experimental study support our hypotheses, demonstrating that the KatApp system is at least as useful and reliable as the conventional paper-based tool for triaging in disasters involving MCIs. The advantages of a digital triage tool—such as automatic forwarding of information to a shared data source and central dashboard, collection of real-time data, tracking of patients’ geolocations, and gathering of complex data to optimize rescue operations—are clearly evident. In addition to our quantitative results, we observed several additional advantages in our study. During the early implementations of the KatApp and in the experimental study, participants did not receive extensive training before using the system. Instead, they were given only a brief introduction right before the training began. This highlights the overall usability of the KatApp system.

Furthermore, qualitative feedback from the command posts after the training sessions confirmed that the information presented in the dashboard significantly simplified their work. In particular, the real-time overview of patients along with their triage categories, as well as the map displaying patient locations, was highly appreciated.

### Next Steps and Further Adaptions

The next steps will include enhancing interconnectivity between smartphones when mobile networks are unavailable and incorporating emergency resources such as ambulances, helicopters, and hospitals into the backend. We also plan to conduct final tests in realistic scenarios, including the use of full personal protective equipment and simulated body fluids. Following these tests, we will initiate a pilot phase in real, nonsimulated settings with the goal of introducing the live system as quickly as possible.

### Comparison With Prior Work

To our knowledge, this is the first paper addressing the challenges of current rescue operations in mass casualty disasters through the development, implementation, and experimental evaluation of a mobile triage app compared with a conventional paper-based tool. A systematic review of triage apps for health emergencies [34] indicates that there have been previous attempts to develop similar approaches. For example, in 2014, a research group developed a mobile-based system to support triage in MCIs, incorporating a Cox proportional hazard model to present patients’ survival curves and aid in triage and transportation decisions [35]. Another approach involved using smart glasses along with a specific Android app to assist in triage during MCIs, which was evaluated in a randomized controlled simulation trial [36]. The TRIAGIST mobile app [37,38] was developed as a low-cost system that dispatch centers or rescue teams in Thailand could use for developmental purposes. However, none of these approaches have been further implemented for use in disaster medicine to date. Additionally, there has been a decline in studies focused on apps for catastrophe triage in recent years, and the few existing triage apps on the market that are based on scientific and academic research often lack free access [34]. In response to the call for an accessible app that provides patient geolocation and assists rescue teams by automatically applying a triage algorithm [34], our KatApp system aims to address a significant gap in the field. This contribution is of great practical importance for improving triage processes in disaster scenarios.

### Limitations

In our study, we utilized patient cards instead of real patients or actors, which may limit the external validity of our findings. Our primary goal was to compare both triage tools within a standardized procedure and analyze a large number of triages, resulting in a total of 2280 triages. Involving 60 actors would have been more costly with only marginal additional benefit. The KatApp still needs to be evaluated in real disaster operations or large-scale simulations. Regarding the within-subjects design, we acknowledge that order effects, such as practice or fatigue, cannot be completely ruled out. To address this, we counterbalanced the tool order and included it as a between-subjects factor in our MANOVA. We also conducted a second repeated-measures MANOVA with measurement time points as a within-subjects factor. Our analyses revealed no significant influence of order on the primary outcomes. While practice effects were observed, participants consistently performed better in terms of time and accuracy with the KatApp. Despite these order effects, we chose a within-subjects design for its greater statistical power, better control over individual differences, and smaller sample size requirement. However, within-subjects designs may also make it easier for participants to infer the hypotheses, potentially distorting results due to heightened expectations. Finally, some assumptions for our statistical analyses, such as the normal distribution of variables, were partially violated. However, MANOVA is generally considered robust to such violations [39]. Although the overall missing data rate was high (10/48, 21%), we still reported large effect sizes in our results.

### Conclusions

In this paper, we present research that addresses the weaknesses and challenges of the current triage process in MCIs through the development, implementation, and evaluation of a mobile system called KatApp.

We demonstrated that the KatApp is a mobile triage app designed for disaster medicine that not only matches the performance of the conventional paper-based tool but may even surpass it in terms of efficiency and usability. This advancement highlights the potential of digitalization to optimize processes in disaster medicine, ultimately leading to the possibility of saving more lives. While it is clear that this novel approach reduces time-consuming and error-prone manual tasks, we are currently unable to fully quantify the impact of the KatApp system on efficiency in real disaster situations, as it has only been implemented in simulated training scenarios thus far. Further research and refinement of this approach are needed to address this gap.

## Appendix

Multimedia Appendix 1 Experiment overview picture.

Multimedia Appendix 2 Experiment overview film.

Multimedia Appendix 3 Patient cards.

Multimedia Appendix 4 Paper-based casualty cards.

Multimedia Appendix 5 KatApp mock-ups.

Multimedia Appendix 6 German version of the User Experience Questionnaire as used in our study with English translations.

Multimedia Appendix 7 Kendall tau correlations between age and the outcome variables to check the conditions for a multivariate analysis of covariance.

Multimedia Appendix 8 TIDieR (Template for Intervention Description and Replication) checklist.

Multimedia Appendix 9 Post hoc univariate analysis of variance comparison between the paper-based tool and the KatApp (N=38).

## Abbreviations

AWS: Amazon Web Services
EMT: Emergency Medical Technician
MANOVA: multivariate analysis of variance
MCI: mass casualty incident
mSTART: modified Simple Triage and Rapid Treatment
REST: Representational State Transfer
START: Simple Triage and Rapid Treatment
TIDieR: Template for Intervention Description and Replication
UEQ: User Experience Questionnaire

## Data Availability

The data sets generated and analyzed during this study are available from the corresponding author upon reasonable request. The software code for the KatApp cannot be made publicly available. However, interested researchers may inquire about specific aspects of the system’s functionality and architecture. We are committed to ensuring that the data supporting the findings of this study are accessible to the scientific community.
