# Supplementary figures and images for: Evaluation of an App-Based Mobile Triage System for Mass Casualty Incidents: Within-Subjects Experimental Study
Source: J Med Internet Res. 2024 Nov 21;26:e65728. doi: 10.2196/65728 (PMC11621716; doi:10.2196/65728)

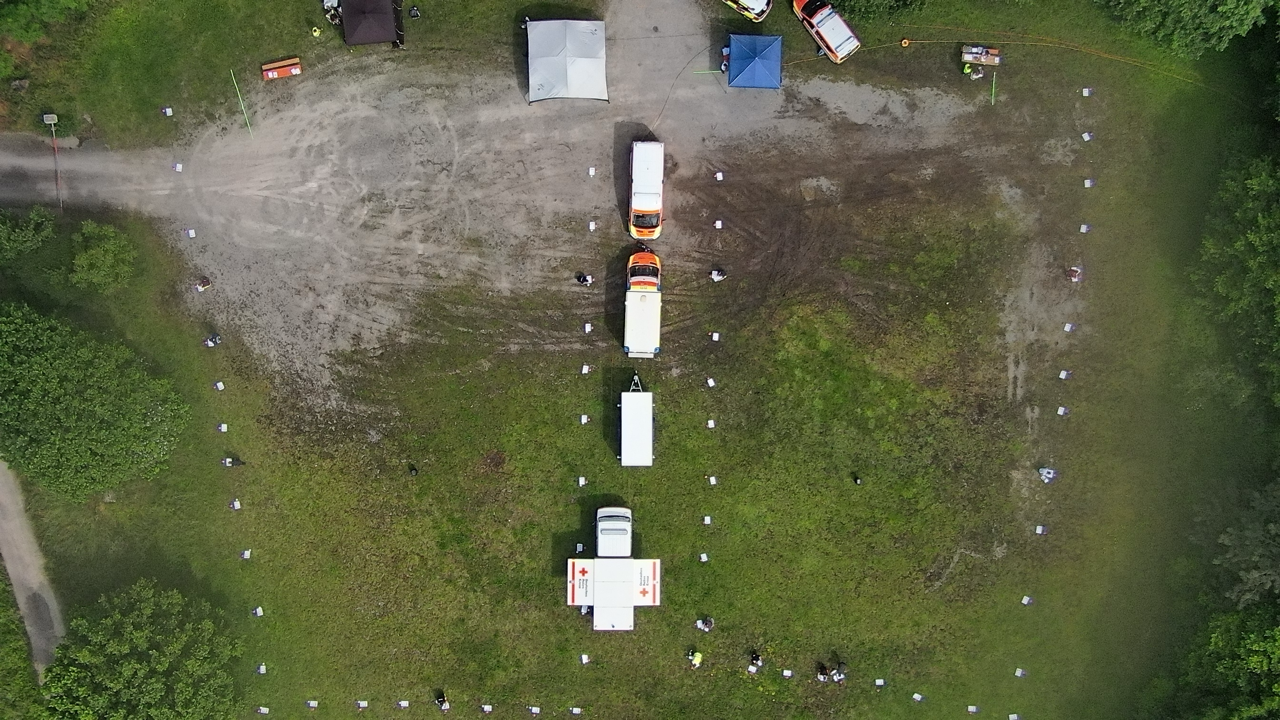

Supplement: Multimedia Appendix 1 [file jmir_v26i1e65728_app1.png]
