# Supplementary material for: Evaluation of an App-Based Mobile Triage System for Mass Casualty Incidents: Within-Subjects Experimental Study
Source: J Med Internet Res. 2024 Nov 21;26:e65728. doi: 10.2196/65728 (PMC11621716; doi:10.2196/65728)
Supplement: Multimedia Appendix 3 [file jmir_v26i1e65728_app3.pdf]

Ali Khan (m)

Ali, ein Geografie-Student, geriet in Panik, als die Situation eskalierte. Auf der Flucht kugelte er sich die Schulter aus und brach sich den rechten Fuß. Er kann nicht mehr aufstehen.

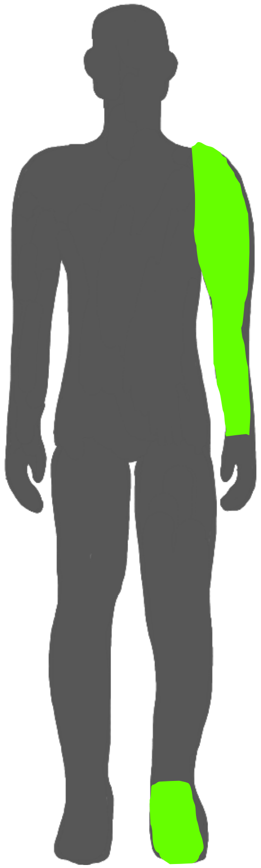

**GCS** (Glasgow-Koma-Skala)<br>Folgt Aufforderungen adäquat	15<br>Ja
**AF** (Atemfrequenz pro Minute)	15
**RRsys** (systolischer Blutdruck in mmHg)	150
Radialispuls tastbar<br>Rekapillarisierungszeit	Ja<br>2sec
**Gehfähig** (Kann der Patient gehen?)	Nein
**Alter** (Alter des Patienten)	52

Dislokation Arm	2/6
Fraktur Fuß	2/6
-	-

Injury Severity (AIS)

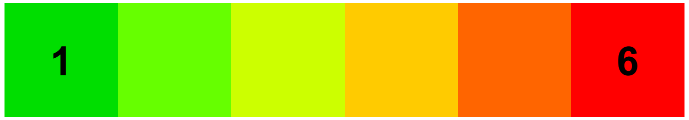

1 gering, 2 moderat, 3 ernst, 4 schwer, 5 kritisch, 6 tödlich

Patienten-ID: **001**

Nina Weber (w)

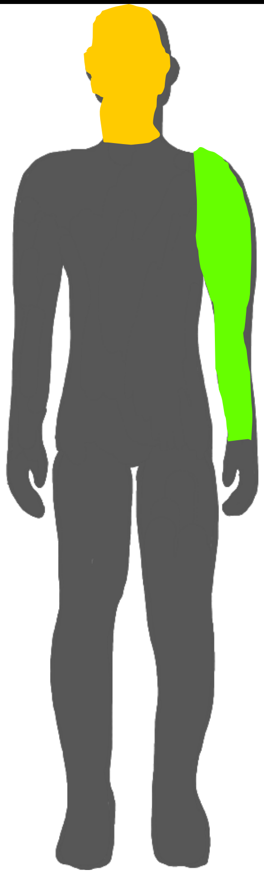

**GCS** (Glasgow-Koma-Skala)<br>Folgt Aufforderungen adäquat	12
**AF** (Atemfrequenz pro Minute)<br>Atemweg frei	30
**RRsys** (systolischer Blutdruck in mmHg)	87
Radialispuls tastbar<br>Rekapillarisierungszeit	Nein<br>4sec
**Gehfähig** (Kann der Patient gehen?)	Nein
**Alter** (Alter des Patienten)	52

Explosionsverletzung Arm	2/6
Schädelfraktur	4/6
-	-

Nina, eine Anwältin, befand sich mit ihrer Familie auf dem Public Viewing, um das Spiel zu genießen. Durch die Explosion erlitt sie zwar nur eine moderate Verletzung am Arm, jedoch durch den anschließenden Sturz eine schwere Schädelfraktur.

Injury Severity (AIS)

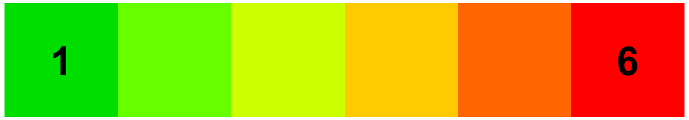

1 gering, 2 moderat, 3 ernst, 4 schwer, 5 kritisch, 6 tödlich

Patienten-ID: **002**

Sofia Fernandez (w)

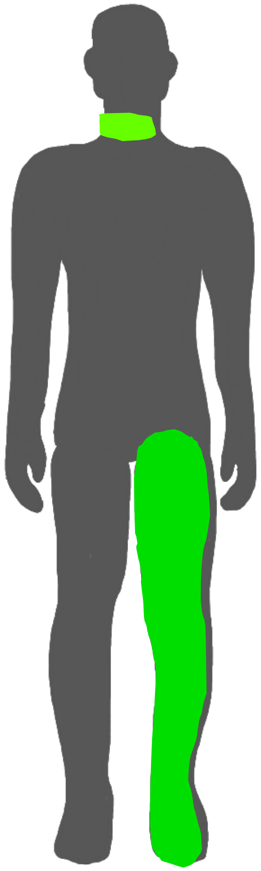

Injury Severity (AIS)

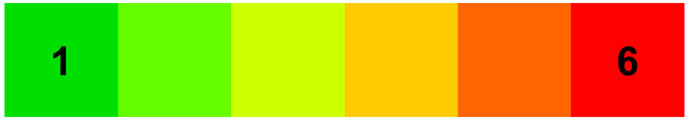

1 gering, 2 moderat, 3 ernst, 4 schwer, 5 kritisch, 6 tödlich

**GCS** (Glasgow-Koma-Skala)<br>Folgt Aufforderungen adäquat	15<br>Ja
**AF** (Atemfrequenz pro Minute)	16
**RRsys** (systolischer Blutdruck in mmHg)	183
Radialispuls tastbar<br>Rekapillarisierungszeit	Ja<br>2sec
**Gehfähig** (Kann der Patient gehen?)	Ja
**Alter** (Alter des Patienten)	2

Verstauchung/Zerrung Bein	1/6
Verstauchung/Zerrung Nacken	2/6
-	-

Sofia ist eine Austauschstudentin, die in Deutschland Deutsch studiert. Sie war mit ihren Kommilitonen auf dem Public Viewing, um das Spiel zu verfolgen. Sie hat leichte Verletzungen am Bein und Nacken erlitten und ist momentan sehr ängstlich.

Patienten-ID: **003**

Laura Schneider (w)

Laura, eine Bankangestellte, die mir ihrer Familie beim Public Viewing war, zog sich in der Massenpanik verschiedene Verletzungen zu.

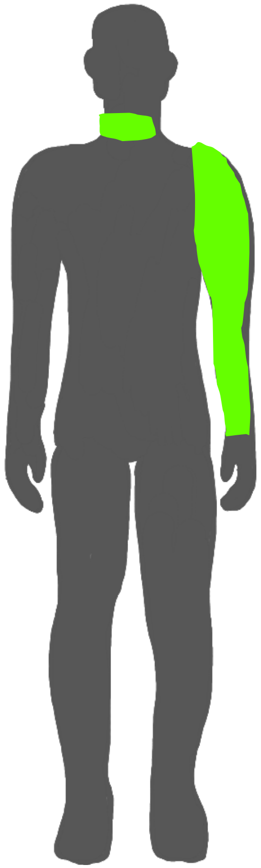

Injury Severity (AIS)

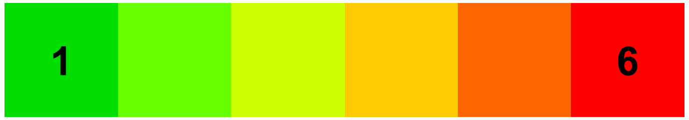

1 gering, 2 moderat, 3 ernst, 4 schwer, 5 kritisch, 6 tödlich

**GCS** (Glasgow-Koma-Skala)<br>Folgt Aufforderungen adäquat	15<br>Ja
**AF** (Atemfrequenz pro Minute)	24
**RRsys** (systolischer Blutdruck in mmHg)	145
Radialispuls tastbar<br>Rekapillarisierungszeit	Ja<br>2sec
**Gehfähig** (Kann der Patient gehen?)	Ja
**Alter** (Alter des Patienten)	59

Dislokation Arm	2/6
Verstauchung/Zerrung Nacken	2/6
-	-

Patienten-ID: **004**

Julia Fischer (w)

Julia, eine Physiotherapeutin, zeigte großen Einsatz und Mitgefühl, als die Katastrophe passierte. Auf der Flucht vor dem Angriff prellte sie sich beide Oberarme und ist sehr durcheinander.

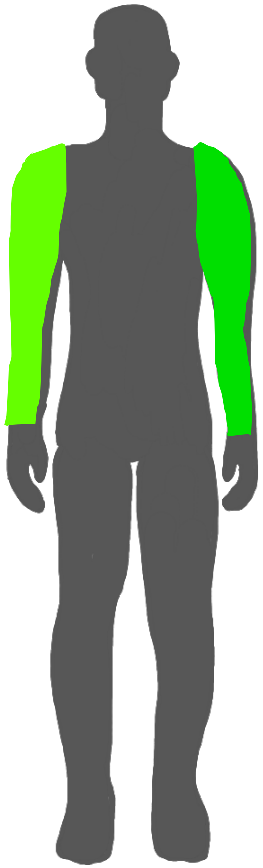

Injury Severity (AIS)

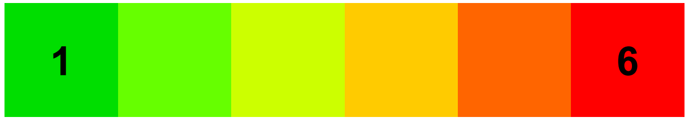

1 gering, 2 moderat, 3 ernst, 4 schwer, 5 kritisch, 6 tödlich

**GCS** (Glasgow-Koma-Skala)<br>Folgt Aufforderungen adäquat	15<br>Ja
**AF** (Atemfrequenz pro Minute)	12
**RRsys** (systolischer Blutdruck in mmHg)	100
Radialispuls tastbar<br>Rekapillarisierungszeit	Ja<br>2sec
**Gehfähig** (Kann der Patient gehen?)	Ja
**Alter** (Alter des Patienten)	24

Prellung Arm rechts	2/6
Prellung Arm links	1/6
-	-

Patienten-ID: **005**

Alessio Rossi (m)

Alessio ist ein Architekt, der für ein Projekt nach Deutschland gereist ist. Er hat Schmerzen im Arm, nachdem er darauf gefallen war.

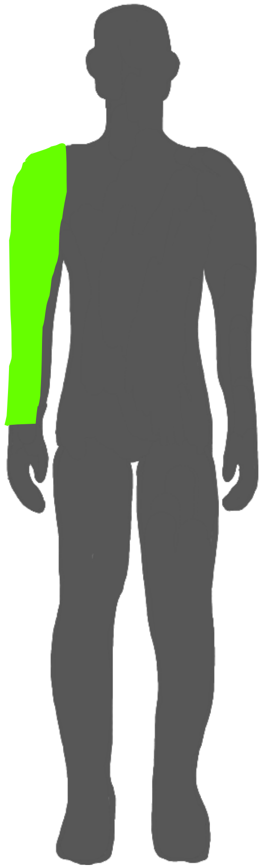

**GCS** (Glasgow-Koma-Skala)<br>Folgt Aufforderungen adäquat	15<br>Ja
**AF** (Atemfrequenz pro Minute)	19
**RRsys** (systolischer Blutdruck in mmHg)	159
Radialispuls tastbar<br>Rekapillarisierungszeit	Ja<br>2sec
**Gehfähig** (Kann der Patient gehen?)	Ja
**Alter** (Alter des Patienten)	52

Dislokation Arm	2/6
-	-
-	-

Injury Severity (AIS)

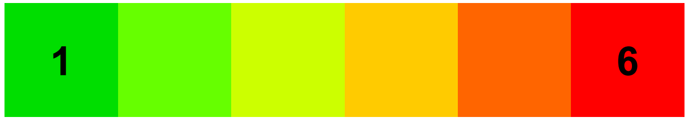

1 gering, 2 moderat, 3 ernst, 4 schwer, 5 kritisch, 6 tödlich

Patienten-ID: **006**

Maria Weber (w)

Maria, eine passionierte Krankenschwester, handelte sofort, als der Angriff begann. Durch die Explosion erlitt sie Verletzungen an den Armen sowie eine Quetschverletzung am Fuß und ist nun zu schwach, um aufzustehen.

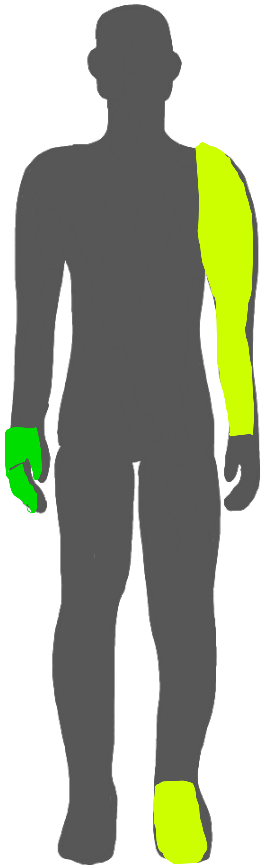

Injury Severity (AIS)

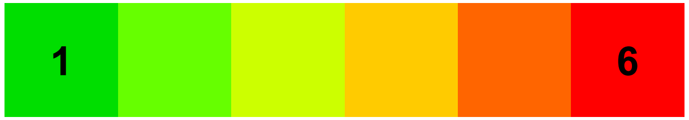

1 gering, 2 moderat, 3 ernst, 4 schwer, 5 kritisch, 6 tödlich

**GCS** (Glasgow-Koma-Skala)<br>Folgt Aufforderungen adäquat	15<br>Ja
**AF** (Atemfrequenz pro Minute)	19
**RRsys** (systolischer Blutdruck in mmHg)	156
Radialispuls tastbar<br>Rekapillarisierungszeit	Ja<br>2sec
**Gehfähig** (Kann der Patient gehen?)	Nein
**Alter** (Alter des Patienten)	37

Explosionsverletzung Arm	3/6
Explosionsverletzung Finger	1/6
Quetschung Fuß	3/6

Patienten-ID: **007**

Luca Costa (m)

Luca, ein junger Sportstudent, war auf dem Public Viewing, um das Spiel mit seinen Freunden zu erleben. Als der Angriff begann, flüchtete er, wurde jedoch erfasst und zog sich kritische Kopf- sowie weitere Verletzungen zu.

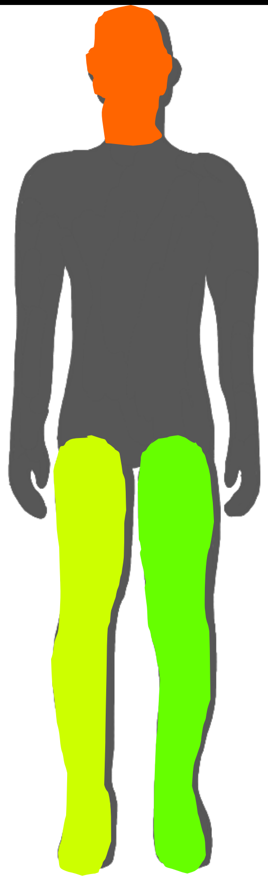

Injury Severity (AIS)

1 … 6

1 gering, 2 moderat, 3 ernst, 4 schwer, 5 kritisch, 6 tödlich

**GCS** (Glasgow-Koma-Skala)<br>Folgt Aufforderungen adäquat	11<br>Nein
**AF** (Atemfrequenz pro Minute)<br>Atemweg frei	32
**RRsys** (systolischer Blutdruck in mmHg)	74
Radialispuls tastbar<br>Rekapillarisierungszeit	Nein<br>5sec
**Gehfähig** (Kann der Patient gehen?)	Nein
**Alter** (Alter des Patienten)	19
Quetschung Kopf	5/6
Quetschung Bein	3/6
Dislokation Bein	1/6

Patienten-ID: **008**

Sebastian Schmidt (m)

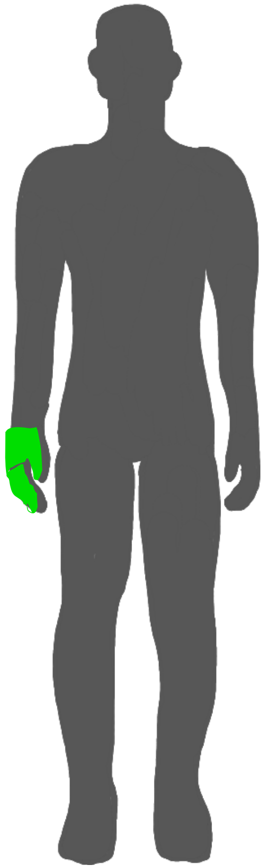

Injury Severity (AIS)

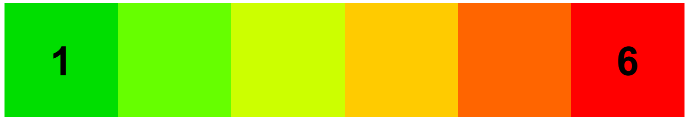

1 gering, 2 moderat, 3 ernst, 4 schwer, 5 kritisch, 6 tödlich

**GCS** (Glasgow-Koma-Skala)<br>Folgt Aufforderungen adäquat	15<br>Ja
**AF** (Atemfrequenz pro Minute)	14
**RRsys** (systolischer Blutdruck in mmHg)	127
Radialispuls tastbar<br>Rekapillarisierungszeit	Ja<br>2sec
**Gehfähig** (Kann der Patient gehen?)	Ja
**Alter** (Alter des Patienten)	60

Verstauchung/Zerrung Hand/Finger	1/6
-	-
-	-

Sebastian ist ein pensionierter Ingenieur. Er war mit seiner Frau auf dem Public Viewing, um das Spiel zu genießen. Er hat sich in der Panik die Hand an einer umgefallenen Bank angestoßen.

Patienten-ID: **009**

Amir Khan (m)

Amir ist ein Ingenieur, der in Deutschland lebt. Er war auf dem Public Viewing, um das Spiel mit seinen Freunden zu sehen. Er hat sich eine schwere Quetschverletzung des Kopfes zugezogen.

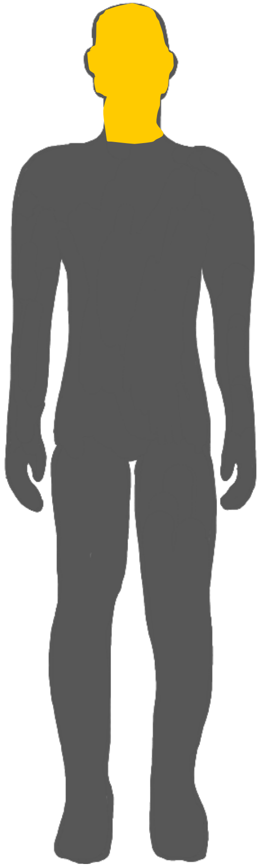

**GCS** (Glasgow-Koma-Skala)<br>Folgt Aufforderungen adäquat	14<br>Ja
**AF** (Atemfrequenz pro Minute)<br>Atemweg frei	28
**RRsys** (systolischer Blutdruck in mmHg)	92
Radialispuls tastbar<br>Rekapillarisierungszeit	Ja<br>2sec
**Gehfähig** (Kann der Patient gehen?)	Nein
**Alter** (Alter des Patienten)	60

Quetschung Kopf	4/6
-	-
-	-

Injury Severity (AIS)

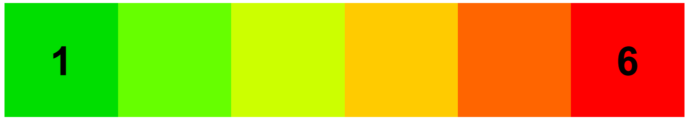

1 gering, 2 moderat, 3 ernst, 4 schwer, 5 kritisch, 6 tödlich

Patienten-ID: **010**

Lena Richter (w)

Lena, eine junge Mathematikstudentin, war auf dem Public Viewing, um den Abend mit ihren Mitbewohnern zu verbringen. Als die Situation eskalierte, wurde sie vom Fahrzeug erfasst und schlug hart auf eine Bordsteinkante auf. Nun hat sie Schwierigkeiten beim Atmen und spürt die Beine nicht mehr.

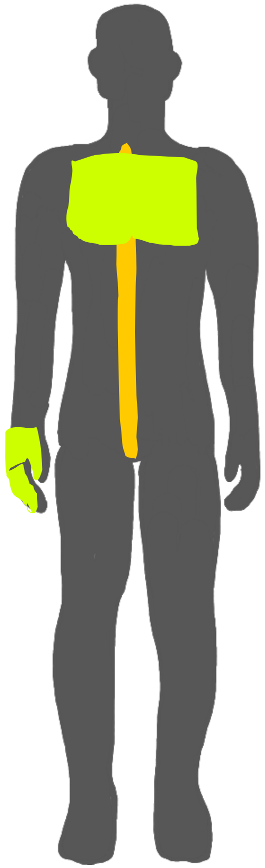

**GCS** (Glasgow-Koma-Skala)<br>Folgt Aufforderungen adäquat	12<br>Nein
**AF** (Atemfrequenz pro Minute)	32
**RRsys** (systolischer Blutdruck in mmHg)	85
Radialispuls tastbar<br>Rekapillarisierungszeit	Nein<br>4sec
**Gehfähig** (Kann der Patient gehen?)	Nein
**Alter** (Alter des Patienten)	23

Fraktur Hand/Finger	3/6
Fraktur Rippen	3/6
Fraktur Wirbelsäule	4/6

Injury Severity (AIS)

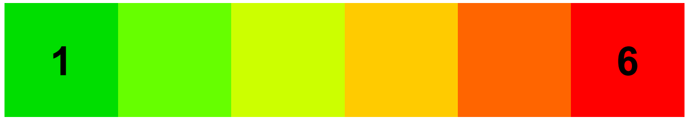

1 gering, 2 moderat, 3 ernst, 4 schwer, 5 kritisch, 6 tödlich

Patienten-ID: **011**

Henrik Jensen (m)

Henrik ist ein Geschäftsmann, der auf Geschäftsreise in Deutschland ist. Er war auf dem Public Viewing, um sich zu entspannen und das Spiel zu sehen. Er hat sich auf der Flucht vor dem Attentäter die Füße und den Arm verstaucht.

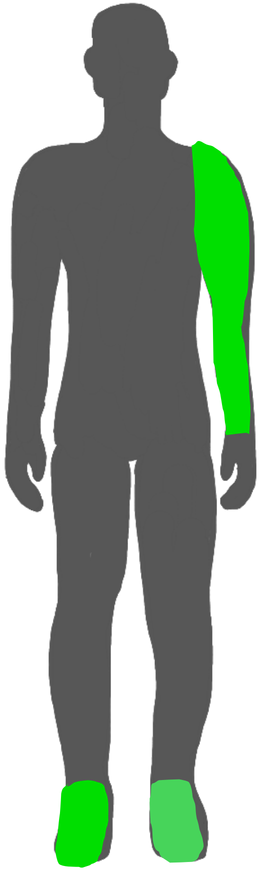

**GCS** (Glasgow-Koma-Skala)<br>Folgt Aufforderungen adäquat	13<br>Ja
**AF** (Atemfrequenz pro Minute)	15
**RRsys** (systolischer Blutdruck in mmHg)	168
Radialispuls tastbar<br>Rekapillarisierungszeit	Ja<br>2sec
**Gehfähig** (Kann der Patient gehen?)	Ja
**Alter** (Alter des Patienten)	49

Injury Severity (AIS)

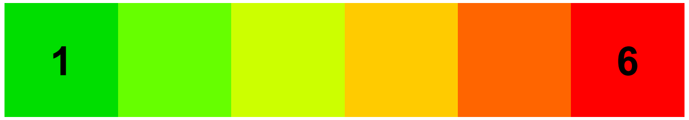

1 gering, 2 moderat, 3 ernst, 4 schwer, 5 kritisch, 6 tödlich

Verstauchung/Zerrung Arm	1/6
Verstauchung/Zerrung Fuß	1/6
Verstauchung/Zerrung Fuß	1/6

Patienten-ID: **012**

Elena Fritz (w)

Elena, eine erfolgreiche Geschäftsfrau, war auf dem Public Viewing, um sich zu entspannen. Als die Tragödie geschah, erlitt sie eine schwere Schädelfraktur und weitere moderate Verletzungen.

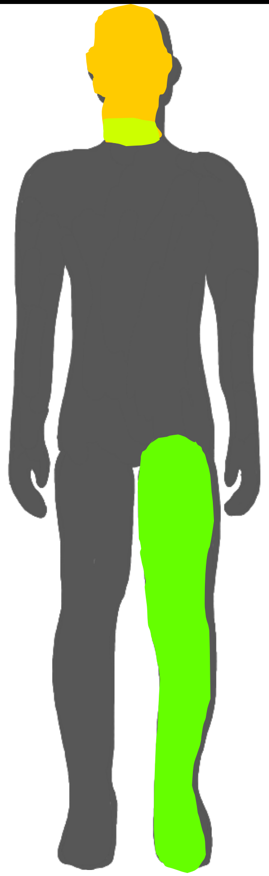

Injury Severity (AIS)

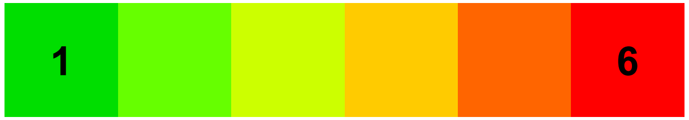

1 gering, 2 moderat, 3 ernst, 4 schwer, 5 kritisch, 6 tödlich

**GCS** (Glasgow-Koma-Skala)<br>Folgt Aufforderungen adäquat	14<br>Ja
**AF** (Atemfrequenz pro Minute)<br>Atemweg frei	33
**RRsys** (systolischer Blutdruck in mmHg)	87
Radialispuls tastbar<br>Rekapillarisierungszeit	Nein<br>4sec
**Gehfähig** (Kann der Patient gehen?)	Nein
**Alter** (Alter des Patienten)	43

Fraktur Schädel	4/6
Verstauchung/Zerrung Bein	2/6
Verstauchung/Zerrung Nacken	3/6

Patienten-ID: **013**

Sarah Bauer (w)

Sarah, eine Kriminalbeamtin aus Deutschland, die sich von ihren Kollegen am Feierabend spontan zum Public Viewing überreden ließ, erlitt bei der Explosion tödliche Verletzungen an Kopf und Bein.

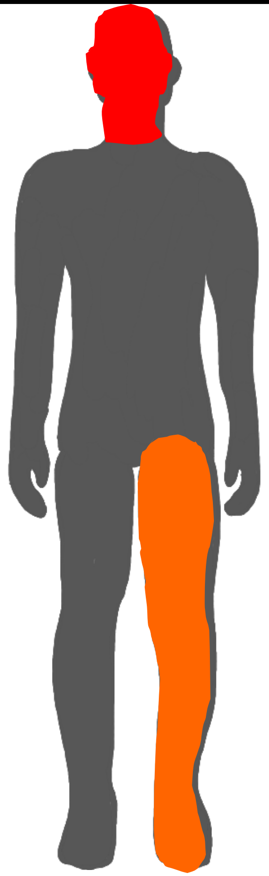

**GCS** (Glasgow-Koma-Skala)<br>Folgt Aufforderungen adäquat	3<br>Nein
**AF** (Atemfrequenz pro Minute)	0
**RRsys** (systolischer Blutdruck in mmHg)	18
Radialispuls tastbar<br>Rekapillarisierungszeit	Nein<br>-
**Gehfähig** (Kann der Patient gehen?)	Nein
**Alter** (Alter des Patienten)	45

Amputation Kopf	6/6
Amputation Bein	5/6
-	-

Injury Severity (AIS)

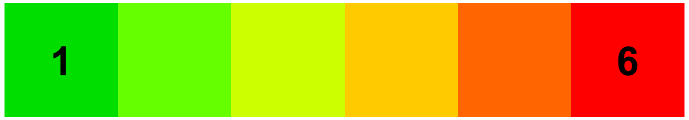

1 gering, 2 moderat, 3 ernst, 4 schwer, 5 kritisch, 6 tödlich

Patienten-ID: **014**

Emilia Müller (w)

Emilia ist eine Krankenschwester, die sich in ihrer Freizeit gerne mit Freunden trifft. Sie war mit ihrer Gruppe auf dem Public Viewing und hat schwere Schnittwunden und eine Unterarmfraktur erlitten. Trotz ihrer eigenen Verletzungen hilft sie anderen Verletzten und versucht, Ruhe zu bewahren.

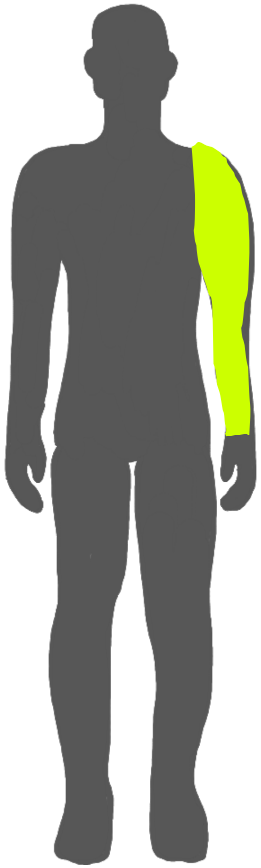

Injury Severity (AIS)

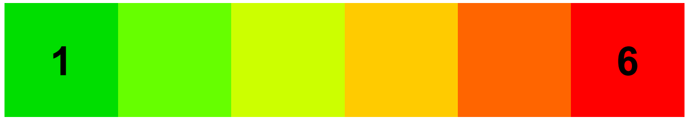

1 gering, 2 moderat, 3 ernst, 4 schwer, 5 kritisch, 6 tödlich

**GCS** (Glasgow-Koma-Skala)<br>Folgt Aufforderungen adäquat	15<br>Ja
**AF** (Atemfrequenz pro Minute)	17
**RRsys** (systolischer Blutdruck in mmHg)	175
Radialispuls tastbar<br>Rekapillarisierungszeit	Ja<br>2sec
**Gehfähig** (Kann der Patient gehen?)	Ja
**Alter** (Alter des Patienten)	29

Fraktur Arm	3/6
-	-
-	-

Patienten-ID: **015**

Lukas Berger (m)

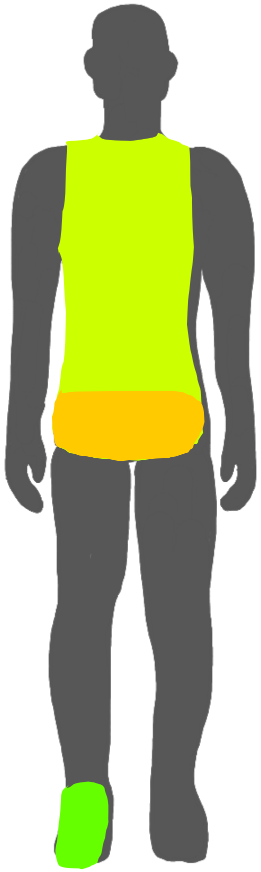

Injury Severity (AIS)

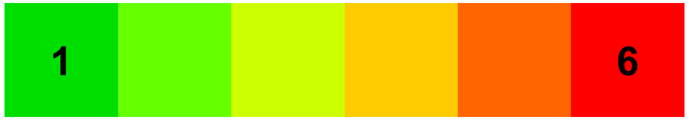

1 gering, 2 moderat, 3 ernst, 4 schwer, 5 kritisch, 6 tödlich

**GCS** (Glasgow-Koma-Skala)<br>Folgt Aufforderungen adäquat	13<br>Ja
**AF** (Atemfrequenz pro Minute)	26
**RRsys** (systolischer Blutdruck in mmHg)	90
Radialispuls tastbar<br>Rekapillarisierungszeit	Ja<br>2sec
**Gehfähig** (Kann der Patient gehen?)	Nein
**Alter** (Alter des Patienten)	27

Quetschung Rücken	3/6
Fraktur Becken/Beckenring	4/6
Verstauchung/Zerrung Fuß	1/6

Lukas ist ein Geschäftsmann aus Deutschland. Er war auf dem Public Viewing, um sich nach einer langen Arbeitswoche zu entspannen. Als der Angriff geschah, versuchte er sofort, Menschen in Sicherheit zu bringen. Dabei erlitt er eine Beckenfraktur, eine Quetschverletzung am Rücken und eine Zerrung am linken Fuß.

Patienten-ID: **016**

Felix Schmitt (m)

Felix, der als Gemeindereferent arbeitete, wurde voll vom Fahrzeug erwischt und durch die Luft geschleudert. Dabei zog er sich tödliche Verletzungen zu.

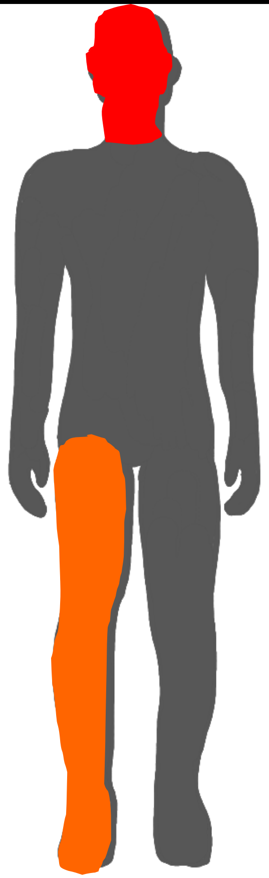

Injury Severity (AIS)

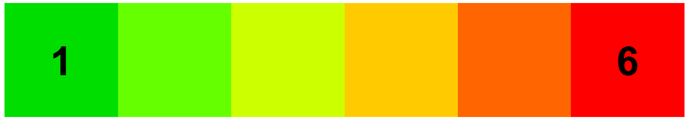

1 gering, 2 moderat, 3 ernst, 4 schwer, 5 kritisch, 6 tödlich

**GCS** (Glasgow-Koma-Skala)<br>Folgt Aufforderungen adäquat	3<br>Nein
**AF** (Atemfrequenz pro Minute)	0
**RRsys** (systolischer Blutdruck in mmHg)	27
Radialispuls tastbar<br>Rekapillarisierungszeit	Nein<br>-
**Gehfähig** (Kann der Patient gehen?)	Nein
**Alter** (Alter des Patienten)	48

Amputation Kopf	6/6
Amputation Bein	5/6
-	-

Patienten-ID: **017**

Benjamin Patel (m)

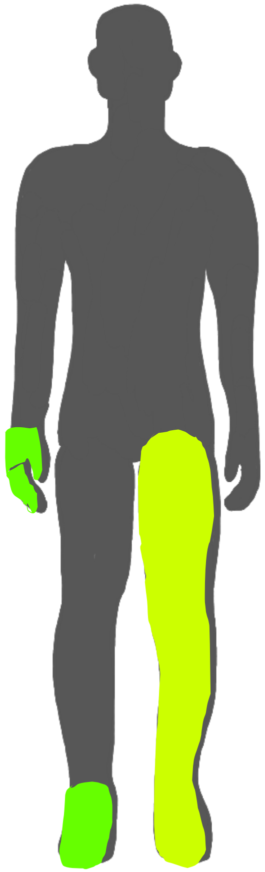

Injury Severity (AIS)

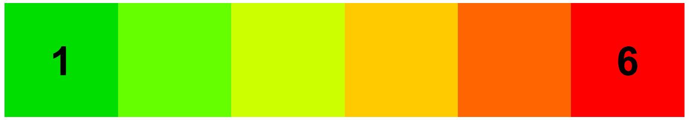

1 gering, 2 moderat, 3 ernst, 4 schwer, 5 kritisch, 6 tödlich

**GCS** (Glasgow-Koma-Skala)<br>Folgt Aufforderungen adäquat	13<br>Ja
**AF** (Atemfrequenz pro Minute)	27
**RRsys** (systolischer Blutdruck in mmHg)	100
Radialispuls tastbar<br>Rekapillarisierungszeit	Ja<br>2sec
**Gehfähig** (Kann der Patient gehen?)	Nein
**Alter** (Alter des Patienten)	42

Explosionsverletzung Bein	3/6
Fraktur Fuß	2/6
Fraktur Hand	2/6

Benjamin ist ein Koch, der in Deutschland ein Restaurant betreibt. Er war auf dem Public Viewing, um sich zu entspannen und das Spiel zu sehen. Er hat sich bei der Explosion des Fahrzeugs eine Verletzung am linken Bein und an der rechten Hand, sowie einen Bruch des rechten Fuß zugezogen.

Patienten-ID: **018**

Tobias Fischer (m)

Tobias, ein Fußballfan, war auf dem Public Viewing, um das Spiel mit seinen Freunden zu sehen. Als die Tragödie geschah, wurde er vom Fahrzeug des Angreifers erfasst und erlitt ein SHT sowie eine Verletzung am rechten Knie.

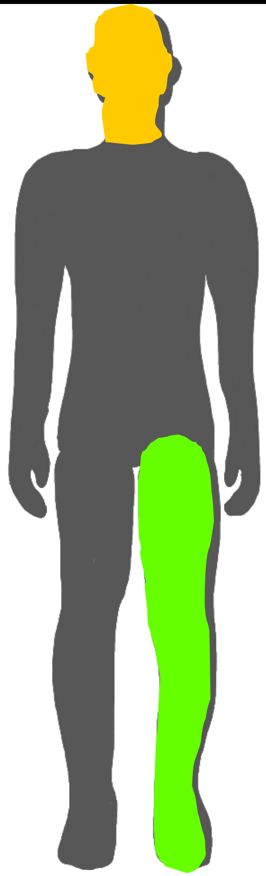

**GCS** (Glasgow-Koma-Skala)<br>Folgt Aufforderungen adäquat	12<br>Nein
**AF** (Atemfrequenz pro Minute)<br>Atemweg frei	34
**RRsys** (systolischer Blutdruck in mmHg)	89
Radialispuls tastbar<br>Rekapillarisierungszeit	Nein<br>4sec
**Gehfähig** (Kann der Patient gehen?)	Nein
**Alter** (Alter des Patienten)	24

Dislokation Bein	2/6
Fraktur Kopf	4/6
-	-

Injury Severity (AIS)

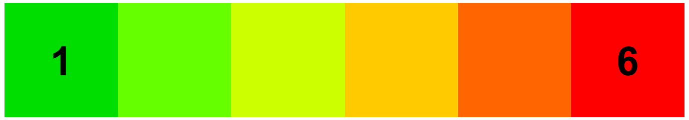

1 gering, 2 moderat, 3 ernst, 4 schwer, 5 kritisch, 6 tödlich

Patienten-ID: **019**

Lea Andersen (w)

Lea ist eine Reisebloggerin, die in Deutschland unterwegs ist. Sie war auf dem Public Viewing, um über die deutsche Fußballkultur zu berichten. Sie hat sich den Oberschenkel gebrochen und benötigt ärztliche Untersuchung.

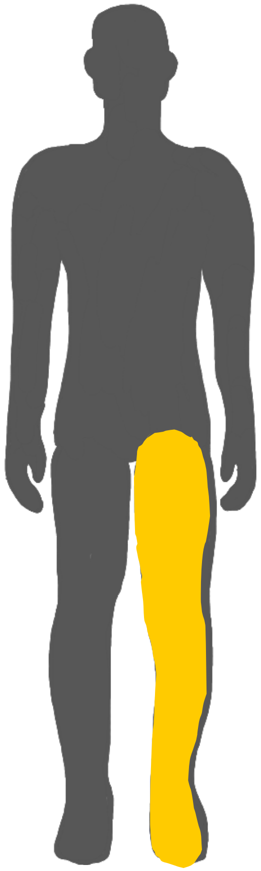

**GCS** (Glasgow-Koma-Skala)<br>Folgt Aufforderungen adäquat	15<br>Ja
**AF** (Atemfrequenz pro Minute)	17
**RRsys** (systolischer Blutdruck in mmHg)	174
Radialispuls tastbar<br>Rekapillarisierungszeit	Ja<br>2sec
**Gehfähig** (Kann der Patient gehen?)	Nein
**Alter** (Alter des Patienten)	33

Fraktur Bein	4/6
-	-
-	-

Injury Severity (AIS)

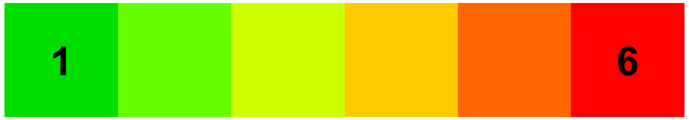

1 gering, 2 moderat, 3 ernst, 4 schwer, 5 kritisch, 6 tödlich

Patienten-ID: **020**

Anna Müller (w)

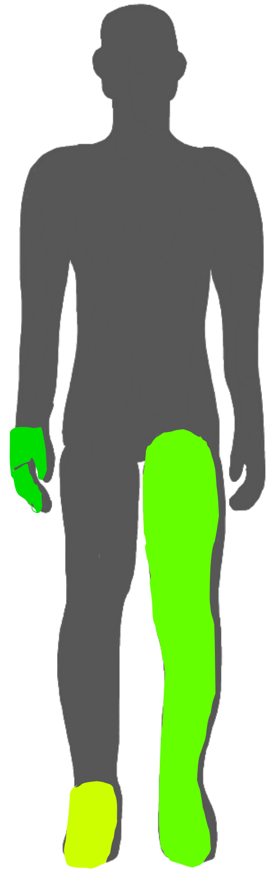

Injury Severity (AIS)

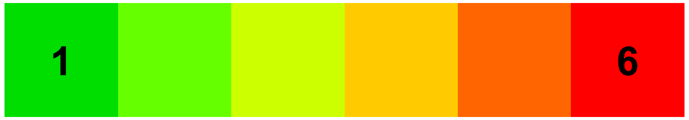

1 gering, 2 moderat, 3 ernst, 4 schwer, 5 kritisch, 6 tödlich

**GCS** (Glasgow-Koma-Skala)<br>Folgt Aufforderungen adäquat	15<br>Ja
**AF** (Atemfrequenz pro Minute)	19
**RRsys** (systolischer Blutdruck in mmHg)	167
Radialispuls tastbar<br>Rekapillarisierungszeit	Ja<br>2sec
**Gehfähig** (Kann der Patient gehen?)	Nein
**Alter** (Alter des Patienten)	47

Quetschung Fuß	3/6
Verstauchung/Zerrung Hand/Finger	1/6
Verstauchung/Zerrung Bein	2/6

Anna ist eine Lehrerin, die das Fußballspiel mit ihren Freunden genießen wollte. Als der LKW in die Menschenmenge fuhr, befand sie sich nahe der Rampe und wurde von umherfliegenden Trümmern getroffen. Sie erlitt mehrere Verletzungen an den Beinen und der Hand und kann nicht aufstehen.

Patienten-ID: **021**

Isabella Lopez (w)

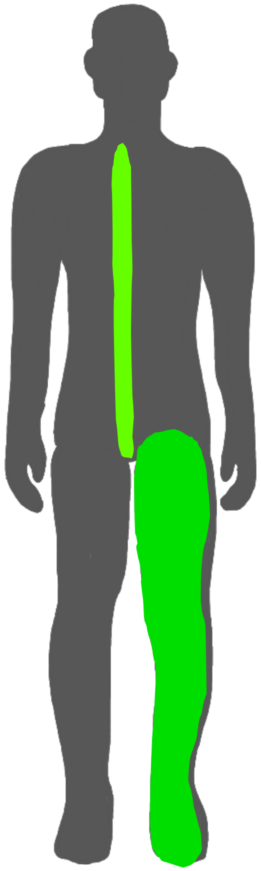

Injury Severity (AIS)

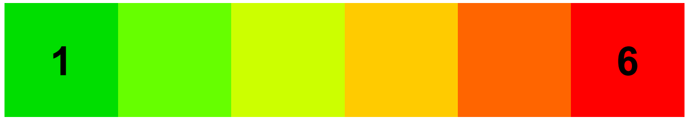

1 gering, 2 moderat, 3 ernst, 4 schwer, 5 kritisch, 6 tödlich

**GCS** (Glasgow-Koma-Skala)<br>Folgt Aufforderungen adäquat	15<br>Ja
**AF** (Atemfrequenz pro Minute)	15
**RRsys** (systolischer Blutdruck in mmHg)	168
Radialispuls tastbar<br>Rekapillarisierungszeit	Ja<br>2sec
**Gehfähig** (Kann der Patient gehen?)	Ja
**Alter** (Alter des Patienten)	65

Verstauchung/Zerrung Bein	1/6
Verstauchung/Zerrung Wirbelsäule	2/6
-	-

Isabella ist eine pensionierte Lehrerin, die Deutschland besucht, um ihre Enkelkinder zu sehen. Sie war mit ihrer Familie auf dem Public Viewing und hat sich bei einem Sturz auf den Rücken eine Verstauchung von Wirbelsäule und dem linken Bein zugezogen. Sie ist gehfähig, aber besorgt um ihre Familie und benötigt Trost.

Patienten-ID: **022**

Mateo Costa (m)

Mateo ist ein Student, der in Deutschland sein Auslandssemester absolviert. Er war mit seinen Mitbewohnern auf dem Public Viewing und hat sich das linke Bein gebrochen sowie den rechten Arm gequetscht. Er ist besorgt um seine Freunde und versucht, sie zu beruhigen.

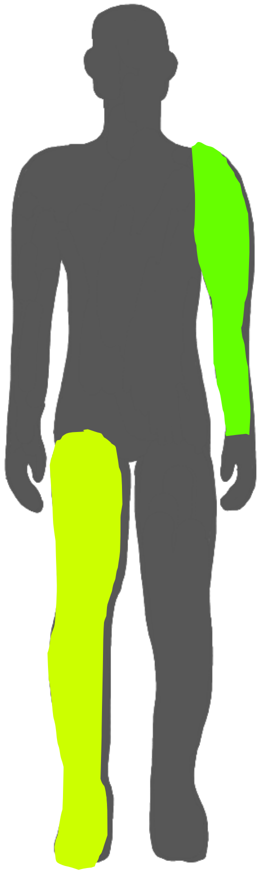

Injury Severity (AIS)

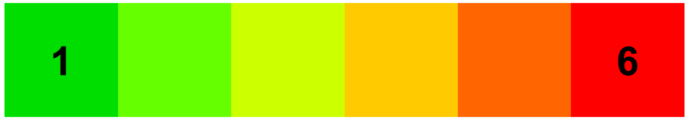

1 gering, 2 moderat, 3 ernst, 4 schwer, 5 kritisch, 6 tödlich

**GCS** (Glasgow-Koma-Skala)<br>Folgt Aufforderungen adäquat	13<br>Ja
**AF** (Atemfrequenz pro Minute)	28
**RRsys** (systolischer Blutdruck in mmHg)	95
Radialispuls tastbar<br>Rekapillarisierungszeit	Ja<br>2sec
**Gehfähig** (Kann der Patient gehen?)	Nein
**Alter** (Alter des Patienten)	20

Quetschung Arm	2/6
Fraktur Bein	3/6
-	-

Patienten-ID: **023**

Max Fischer (m)

Max, ein leidenschaftlicher Fußballfan, bewies großen Einsatz, als die Katastrophe passierte. Er half sofort dabei, Verletzte zu retten und Erste Hilfe zu leisten, dabei stürzte er jedoch unglücklich, erlitt eine Quetschverletzung am Rücken, eine Amputation des rechten Zeigefingers und eine Luxation der Patella.

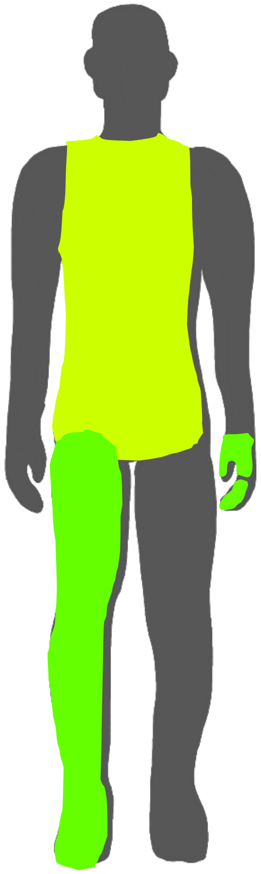

**GCS** (Glasgow-Koma-Skala)<br>Folgt Aufforderungen adäquat	14<br>ja
**AF** (Atemfrequenz pro Minute)	24
**RRsys** (systolischer Blutdruck in mmHg)	95
Radialispuls tastbar<br>Rekapillarisierungszeit	Ja<br>2sec
**Gehfähig** (Kann der Patient gehen?)	Nein
**Alter** (Alter des Patienten)	30

Amputation Hand/Finger<br>Blutung steht	2/6
Quetschung Rücken	3/6
Dislokation Bein	2/6

Injury Severity (AIS)

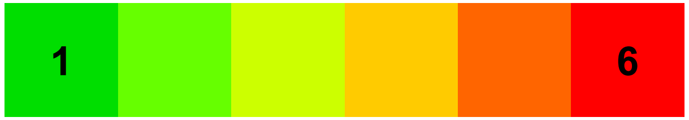

1 gering, 2 moderat, 3 ernst, 4 schwer, 5 kritisch, 6 tödlich

Patienten-ID: **024**

Michael Schmidt (m)

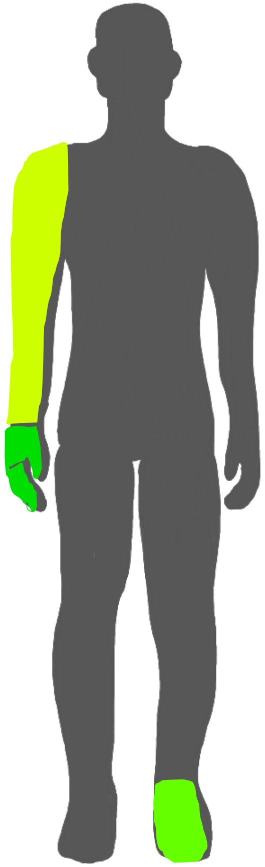

Injury Severity (AIS)

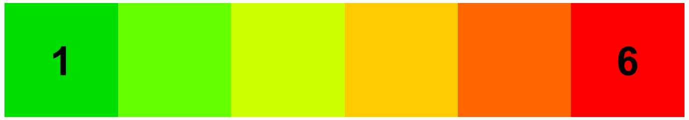

1 gering, 2 moderat, 3 ernst, 4 schwer, 5 kritisch, 6 tödlich

**GCS** (Glasgow-Koma-Skala)<br>Folgt Aufforderungen adäquat	14<br>Ja
**AF** (Atemfrequenz pro Minute)	25
**RRsys** (systolischer Blutdruck in mmHg)	94
Radialispuls tastbar<br>Rekapillarisierungszeit	Ja<br>2sec
**Gehfähig** (Kann der Patient gehen?)	Nein
**Alter** (Alter des Patienten)	29

Fraktur Arm	3/6
Fraktur Fuß	2/6
Verstauchung/Zerrung Hand/Finger	1/6

Michael, ein Ingenieur, war mit seiner Frau auf dem Public Viewing. Als der Angriff begann, schirmte er seine Familie ab und half anderen dabei, sich zu verstecken. Dabei wurde er vom Fahrzeug erfasst als er versuchte, einen Zaun zu überwinden, um Menschen zu retten. Er ist nicht gehfähig.

Patienten-ID: **025**

Jakob Schuster (m)

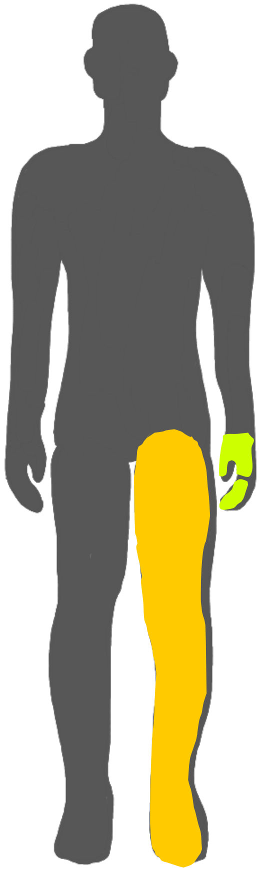

Injury Severity (AIS)

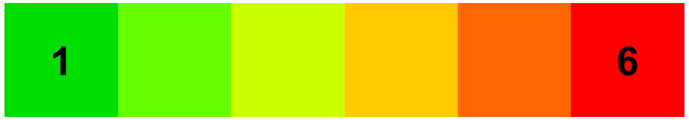

1 gering, 2 moderat, 3 ernst, 4 schwer, 5 kritisch, 6 tödlich

**GCS** (Glasgow-Koma-Skala)<br>Folgt Aufforderungen adäquat	12<br>Nein
**AF** (Atemfrequenz pro Minute)	29
**RRsys** (systolischer Blutdruck in mmHg)	84
Radialispuls tastbar<br>Rekapillarisierungszeit	Nein<br>4sec
**Gehfähig** (Kann der Patient gehen?)	Nein
**Alter** (Alter des Patienten)	45

Amputation Hand/Finger<br>Blutung steht	3/6
Fraktur Bein	4/6

Jakob, ein Gymnasial-Lehrer, genoss den Abend mit seinen Freunden auf dem Public Viewing. Als die Katastrophe passierte, stürzte er in eine Absperrung, teilamputierte dabei den rechten Daumen und brach sich den Oberschenkel geschlossen, welcher nun prall gespannt ist.

Patienten-ID: **026**

Sofia Wagner (w)

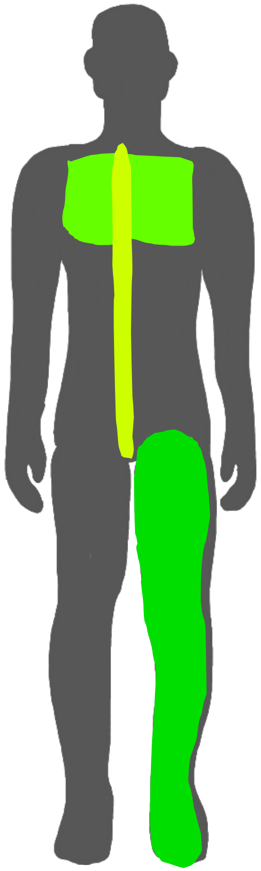

Injury Severity (AIS)

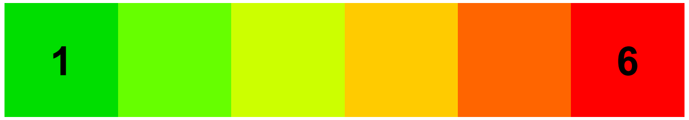

1 gering, 2 moderat, 3 ernst, 4 schwer, 5 kritisch, 6 tödlich

**GCS** (Glasgow-Koma-Skala)<br>Folgt Aufforderungen adäquat	14<br>Ja
**AF** (Atemfrequenz pro Minute)	20
**RRsys** (systolischer Blutdruck in mmHg)	92
Radialispuls tastbar<br>Rekapillarisierungszeit	Ja<br>2sec
**Gehfähig** (Kann der Patient gehen?)	Nein
**Alter** (Alter des Patienten)	62

Fraktur Rippen	2/6
Fraktur Wirbelsäule	3/6
Verstauchung/Zerrung Bein	1/6

Sofia, eine Austauschlehrerin aus Österreich, wollte das Spiel mit ihren deutschen Freunden erleben. Als der LKW auf den Platz raste, war sie in der Nähe der Bühne und wurde von herumfliegenden Trümmern getroffen. Sie hat Schmerzen beim Atmen, im Rücken und im Bein, kann nicht laufen jedoch alles bewegen.

Patienten-ID: **027**

Lara Schulz (w)

Lara ist eine Polizistin, die ihren freien Tag auf dem Public Viewing verbringt. Sie hat eine Brandverletzung am linken Bein und einen Bruch des rechten Fuß davongetragen.

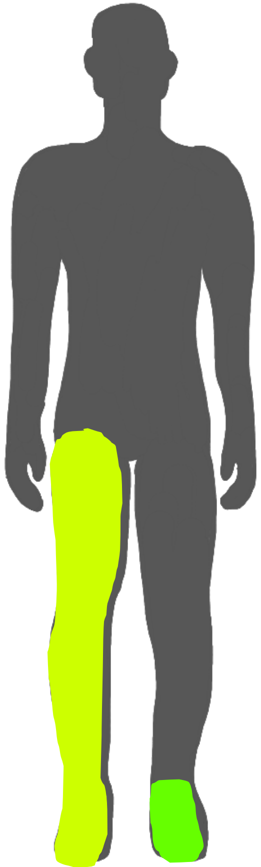

Injury Severity (AIS)

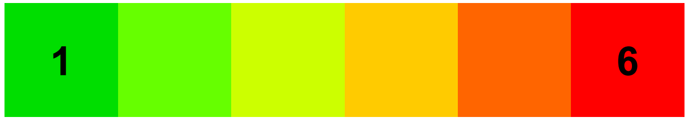

1 gering, 2 moderat, 3 ernst, 4 schwer, 5 kritisch, 6 tödlich

**GCS** (Glasgow-Koma-Skala)<br>Folgt Aufforderungen adäquat	15<br>Ja
**AF** (Atemfrequenz pro Minute)	18
**RRsys** (systolischer Blutdruck in mmHg)	127
Radialispuls tastbar<br>Rekapillarisierungszeit	Ja<br>2sec
**Gehfähig** (Kann der Patient gehen?)	Nein
**Alter** (Alter des Patienten)	34

Brandverletzung Bein	3/6
Fraktur Fuß	2/6
-	-

Patienten-ID: **028**

Nina Ivanova (w)

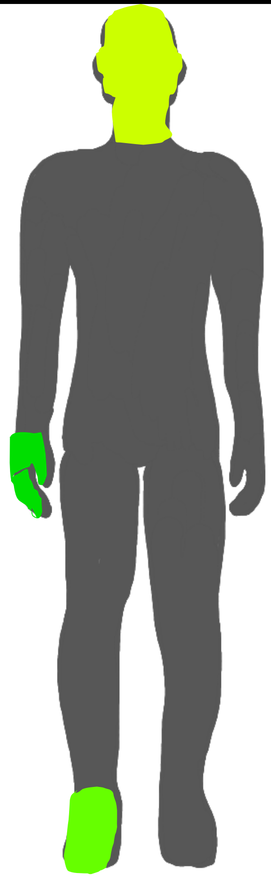

Injury Severity (AIS)

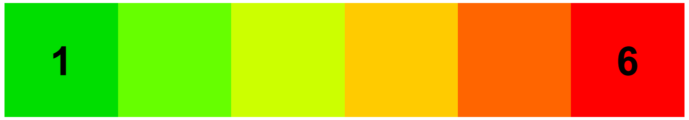

1 gering, 2 moderat, 3 ernst, 4 schwer, 5 kritisch, 6 tödlich

**GCS** (Glasgow-Koma-Skala)<br>Folgt Aufforderungen adäquat	13<br>Ja
**AF** (Atemfrequenz pro Minute)<br>Atemweg frei	23
**RRsys** (systolischer Blutdruck in mmHg)	92
Radialispuls tastbar<br>Rekapillarisierungszeit	Ja<br>2sec
**Gehfähig** (Kann der Patient gehen?)	Nein
**Alter** (Alter des Patienten)	42

Explosionsverletzung Gesicht	3/6
Brandverletzung Fuß	2/6
Verstauchung/Zerrung Hand/Finger	1/6

Nina ist eine Geschäftsfrau, die in Deutschland an einer Konferenz teilnimmt. Sie war auf dem Public Viewing, um sich nach einem langen Arbeitstag zu entspannen. Sie hat durch die Explosion des Fahrzeugs Metall im Gesicht abbekommen, eine Verbrennung am Fuß und eine verstauchte Hand. Die Atemwege sind frei.

Patienten-ID: **029**

David Müller (m)

David, ein Notfallsanitäter, handelte sofort, als die Notlage eintrat. Bei einer Hilfsaktion wurde er jedoch selbst vom Fahrzeug erfasst und erlitt eine moderate Prellung des rechten Beins. Er ist gehfähig.

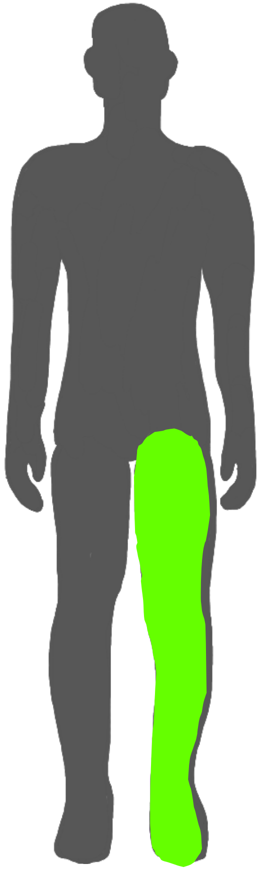

Injury Severity (AIS)

1					6

1 gering, 2 moderat, 3 ernst, 4 schwer, 5 kritisch, 6 tödlich

**GCS** (Glasgow-Koma-Skala)<br>Folgt Aufforderungen adäquat	15<br>Ja
**AF** (Atemfrequenz pro Minute)	16
**RRsys** (systolischer Blutdruck in mmHg)	130
Radialispuls tastbar<br>Rekapillarisierungszeit	Ja<br>2sec
**Gehfähig** (Kann der Patient gehen?)	Ja
**Alter** (Alter des Patienten)	44

Verstauchung/Zerrung Bein	2/6
-	-
-	-

Patienten-ID: **030**

Isabelle Dupont (w)

Isabelle ist eine Französin, die in Deutschland lebt und als Anwältin arbeitet. Sie war auf dem Public Viewing, um das Spiel mit ihren Freunden zu sehen. Neben einigen leichten Verletzungen hat sie sich den Fuß zertrümmert und kann nicht auftreten.

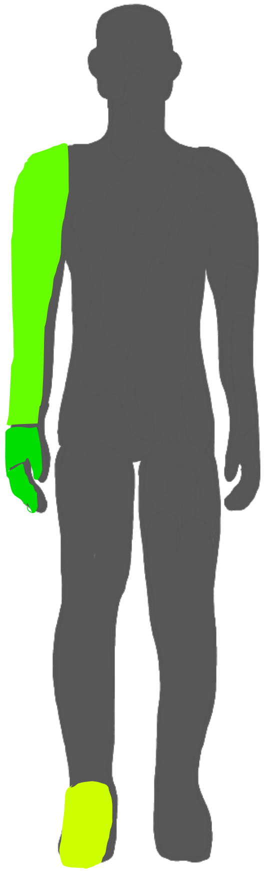

**GCS** (Glasgow-Koma-Skala)<br>Folgt Aufforderungen adäquat	15<br>Ja
**AF** (Atemfrequenz pro Minute)	16
**RRsys** (systolischer Blutdruck in mmHg)	159
Radialispuls tastbar<br>Rekapillarisierungszeit	Ja<br>2sec
**Gehfähig** (Kann der Patient gehen?)	Nein
**Alter** (Alter des Patienten)	41

Quetschung Arm	2/6
Fraktur Fuß	3/6
Verstauchung/Zerrung Hand/Finger	1/6

Injury Severity (AIS)

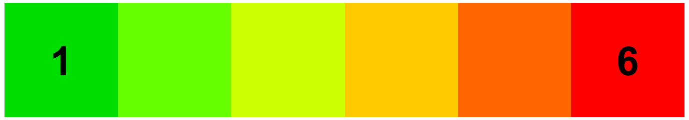

1 gering, 2 moderat, 3 ernst, 4 schwer, 5 kritisch, 6 tödlich

Patienten-ID: **031**

Emma Richter (w)

Emma, eine Erzieherin, war auf dem Public Viewing, um den Abend mit ihren Freunden zu genießen. Sie wurde vom Fahrzeug erfasst und durch die Luft geschleudert. Nun zeigt sie verschiedene Verletzungen.

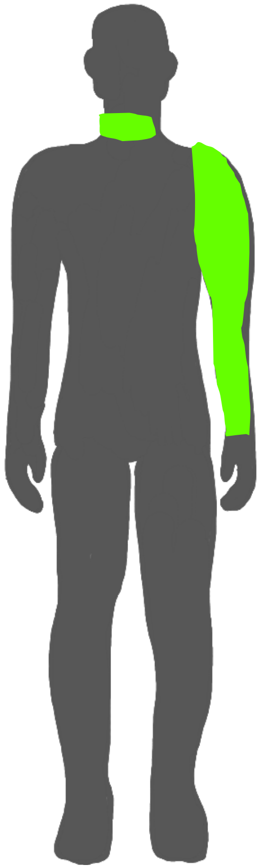

Injury Severity (AIS)

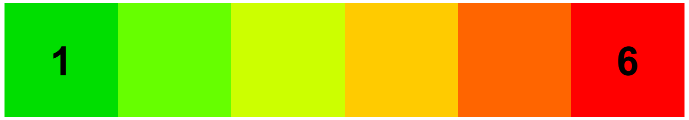

1 gering, 2 moderat, 3 ernst, 4 schwer, 5 kritisch, 6 tödlich

**GCS** (Glasgow-Koma-Skala)<br>Folgt Aufforderungen adäquat	15<br>Ja
**AF** (Atemfrequenz pro Minute)	24
**RRsys** (systolischer Blutdruck in mmHg)	145
Radialispuls tastbar<br>Rekapillarisierungszeit	Ja<br>2sec
**Gehfähig** (Kann der Patient gehen?)	Ja
**Alter** (Alter des Patienten)	58

Dislokation Arm	2/6
Stauchung / Zerrung Nacken	2/6
-	-

Patienten-ID: **032**

Ricardo Santos (m)

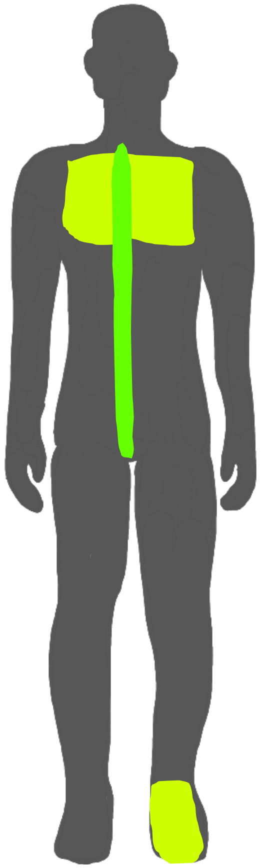

Injury Severity (AIS)

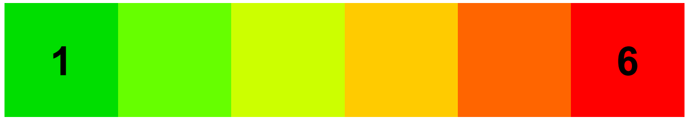

1 gering, 2 moderat, 3 ernst, 4 schwer, 5 kritisch, 6 tödlich

**GCS** (Glasgow-Koma-Skala)<br>Folgt Aufforderungen adäquat	13<br>Ja
**AF** (Atemfrequenz pro Minute)	27
**RRsys** (systolischer Blutdruck in mmHg)	91
Radialispuls tastbar<br>Rekapillarisierungszeit	Ja<br>2sec
**Gehfähig** (Kann der Patient gehen?)	Nein
**Alter** (Alter des Patienten)	28

Quetschung Brust	3/6
Fraktur Fuß	3/6
Verstauchung/Zerrung Wirbelsäule	2/6

Ricardo ist ein brasilianischer Tourist, der Deutschland besucht. Er war auf dem Public Viewing, um die Atmosphäre zu genießen. Er hat sich den Fuß gebrochen und sich dann im Gemenge eine Quetschverletzung an der Brust und eine Stauchung der Wirbelsäule zugezogen.

Patienten-ID: **033**

Elena Fischer (w)

Elena ist eine junge Frau aus Deutschland, die als Grafikdesignerin arbeitet. Beim Public Viewing wollte sie mit ihren Freunden Spaß haben. Nach dem Vorfall versucht sie, Ruhe zu bewahren und trotz gebrochenem Arm anderen zu helfen.

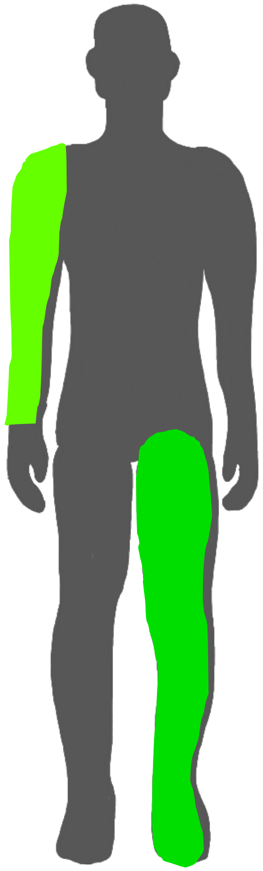

**GCS** (Glasgow-Koma-Skala)<br>Folgt Aufforderungen adäquat	15<br>Ja
**AF** (Atemfrequenz pro Minute)	18
**RRsys** (systolischer Blutdruck in mmHg)	144
Radialispuls tastbar<br>Rekapillarisierungszeit	Ja<br>2sec
**Gehfähig** (Kann der Patient gehen?)	Ja
**Alter** (Alter des Patienten)	30

Fraktur Arm	2/6
Verstauchung/Zerrung Bein	1/6
-	-

Injury Severity (AIS)

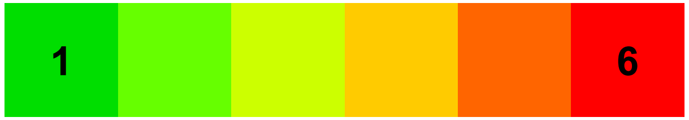

1 gering, 2 moderat, 3 ernst, 4 schwer, 5 kritisch, 6 tödlich

Patienten-ID: **034**

Felix Schäfer (m)

Felix, ein Büroangestellter, war auf dem Public Viewing, um das Spiel mit seinen Freunden zu sehen. Als der Angriff begann, half er dabei, Menschen zu retten. Dann wurde er selbst zum Betroffenen und erlitt verschiedene Verletzungen.

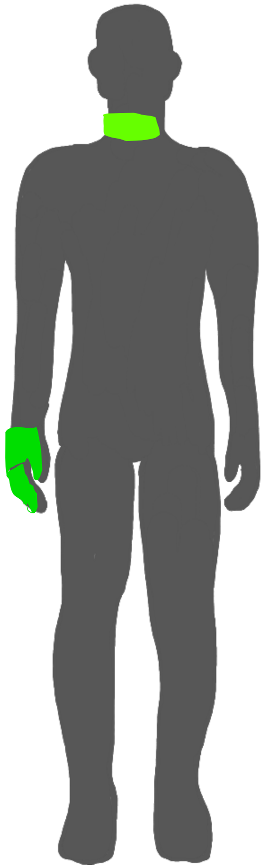

Injury Severity (AIS)

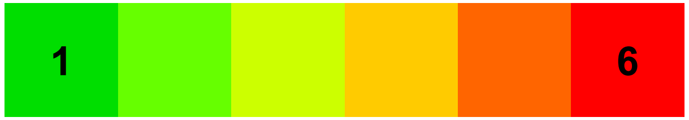

1 gering, 2 moderat, 3 ernst, 4 schwer, 5 kritisch, 6 tödlich

**GCS** (Glasgow-Koma-Skala)<br>Folgt Aufforderungen adäquat	15<br>Ja
**AF** (Atemfrequenz pro Minute)<br>Atemweg frei	12
**RRsys** (systolischer Blutdruck in mmHg)	100
Radialispuls tastbar<br>Rekapillarisierungszeit	Ja<br>2sec
**Gehfähig** (Kann der Patient gehen?)	Ja
**Alter** (Alter des Patienten)	18

Dislokation Hand/Finger	1/6
Verstauchung/Zerrung Neck	2/6
-	-

Patienten-ID: **035**

Sarah Kim (w)

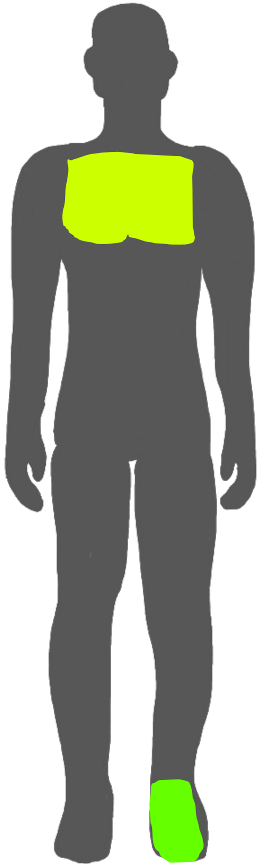

Injury Severity (AIS)

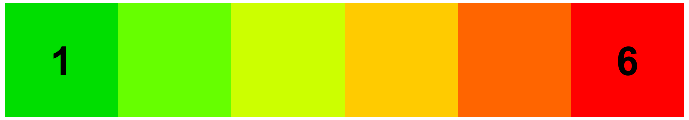

1 gering, 2 moderat, 3 ernst, 4 schwer, 5 kritisch, 6 tödlich

**GCS** (Glasgow-Koma-Skala)<br>Folgt Aufforderungen adäquat	13<br>Ja
**AF** (Atemfrequenz pro Minute)	26
**RRsys** (systolischer Blutdruck in mmHg)	92
Radialispuls tastbar<br>Rekapillarisierungszeit	Ja<br>2sec
**Gehfähig** (Kann der Patient gehen?)	Nein
**Alter** (Alter des Patienten)	35

Brandverletzung Brust	3/6
Dislokation Fuß	2/6
-	-

Sarah ist eine Touristin aus Südkorea, die Deutschland besucht. Sie war auf dem Public Viewing, um die Atmosphäre zu erleben. Sie hat sich ernsthafte Brandverletzungen auf der Brust und eine Luxation des Sprunggelenks zugezogen und kann nicht aufstehen.

Patienten-ID: **036**

Hannah Vogt (w)

Hannah, eine Bademeisterin, genoss den Abend auf dem Public Viewing mit ihren Freunden. Durch den Angreifer wurde sie vor allem kritisch am Kopf verletzt und zog sich eine schwere Beckenfraktur zu.

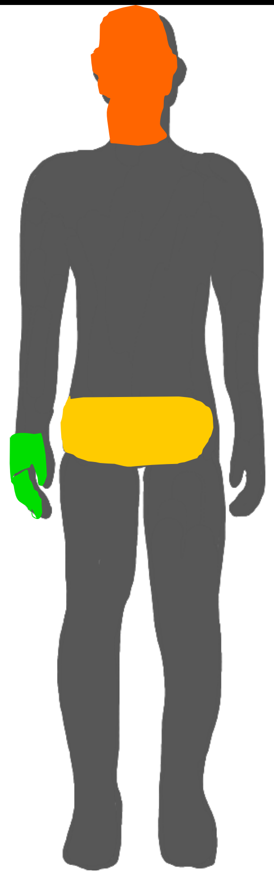

Injury Severity (AIS)

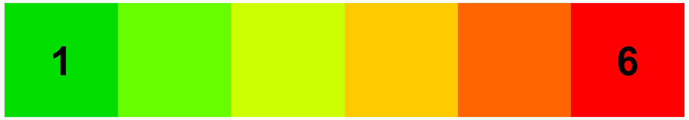

1 gering, 2 moderat, 3 ernst, 4 schwer, 5 kritisch, 6 tödlich

**GCS** (Glasgow-Koma-Skala)<br>Folgt Aufforderungen adäquat	13<br>Nein
**AF** (Atemfrequenz pro Minute)<br>Atemweg frei	32
**RRsys** (systolischer Blutdruck in mmHg)	83
Radialispuls tastbar<br>Rekapillarisierungszeit	Ja<br>4sec
**Gehfähig** (Kann der Patient gehen?)	Nein
**Alter** (Alter des Patienten)	24

Quetschung Hand/Finger	1/6
Quetschung Kopf	5/6
Fraktur Becken/Beckenring	4/6

Patienten-ID: **037**

Mohammad Khan (m)

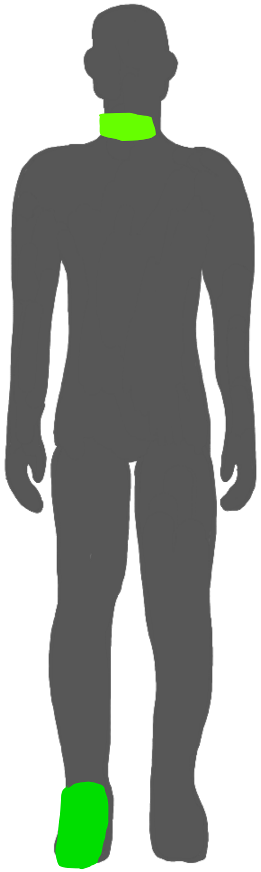

Injury Severity (AIS)

1 … 6

1 gering, 2 moderat, 3 ernst, 4 schwer, 5 kritisch, 6 tödlich

**GCS** (Glasgow-Koma-Skala)<br>Folgt Aufforderungen adäquat	15<br>Ja
**AF** (Atemfrequenz pro Minute)	17
**RRsys** (systolischer Blutdruck in mmHg)	187
Radialispuls tastbar<br>Rekapillarisierungszeit	Ja<br>2sec
**Gehfähig** (Kann der Patient gehen?)	Ja
**Alter** (Alter des Patienten)	50

Verstauchung/Zerrung Fuß	1/6
Verstauchung/Zerrung Nacken	2/6
-	-

Mohammad ist ein Geschäftsmann aus Pakistan, der zu einem Geschäftstreffen in Deutschland war. Er war auf dem Public Viewing, um sich nach einem langen Arbeitstag zu entspannen. Bei der Amokfahrt wurde er leicht verletzt.

Patienten-ID: **038**

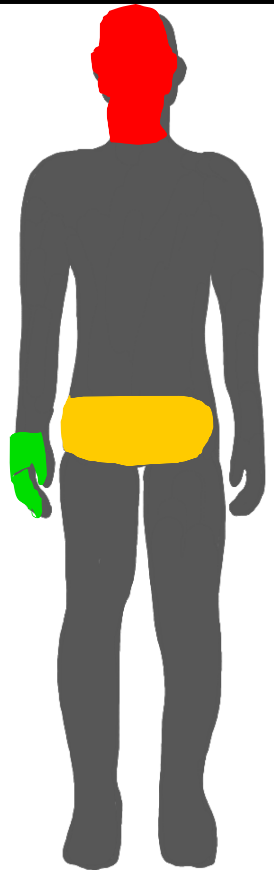

Injury Severity (AIS)

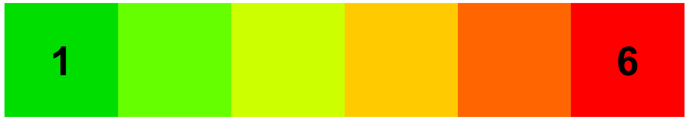

1 gering, 2 moderat, 3 ernst, 4 schwer, 5 kritisch, 6 tödlich

**GCS** (Glasgow-Koma-Skala)<br>Folgt Aufforderungen adäquat	3<br>Nein
**AF** (Atemfrequenz pro Minute)	0
**RRsys** (systolischer Blutdruck in mmHg)	22
Radialispuls tastbar<br>Rekapillarisierungszeit	Nein<br>-
**Gehfähig** (Kann der Patient gehen?)	Nein
**Alter** (Alter des Patienten)	47

Amputation Kopf	6/6
Fraktur Becken/Beckenring	4/6
Dislokation Hand	1/6

David Schmidt (m)

David arbeitet als Bauzeichner in einem Ingenieurbüro. Er war mit seinem Schwager beim Public Viewing, als er voll vom Fahrzeug erwischt wurde und tödliche Verletzungen erlitt.

Patienten-ID: **039**

Paul Hartmann (m)

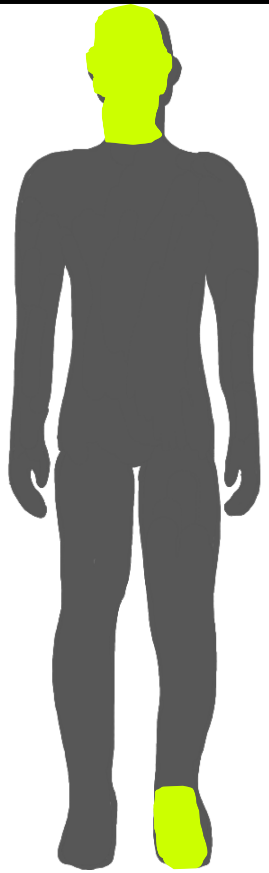

Injury Severity (AIS)

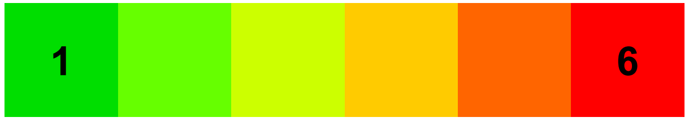

1 gering, 2 moderat, 3 ernst, 4 schwer, 5 kritisch, 6 tödlich

**GCS** (Glasgow-Koma-Skala)<br>Folgt Aufforderungen adäquat	13<br>Nein
**AF** (Atemfrequenz pro Minute)<br>Atemweg frei	36
**RRsys** (systolischer Blutdruck in mmHg)	91
Radialispuls tastbar<br>Rekapillarisierungszeit	Ja<br>2sec
**Gehfähig** (Kann der Patient gehen?)	Nein
**Alter** (Alter des Patienten)	25

Amputation Fuß (Blutung steht)	3/6
Explosionsverletzung Gesicht	3/6
-	-

Paul, ein Rettungssanitäter, war auf dem Public Viewing, um das Spiel mit seiner Familie zu erleben. Dabei zog er sich ernste Verletzungen im Gesicht sowie eine Teilamputation des rechten Fuß zu.

Patienten-ID: **040**

Jim Wagner (m)

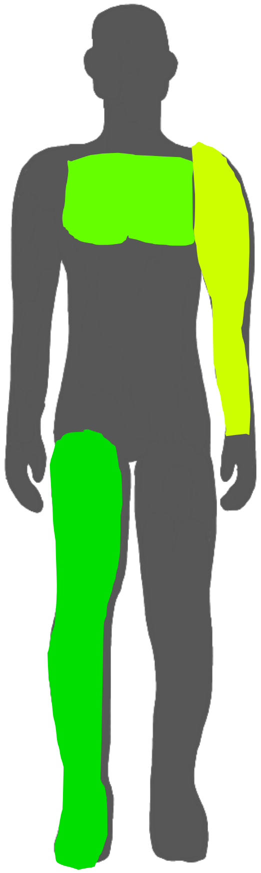

**GCS** (Glasgow-Koma-Skala)<br>Folgt Aufforderungen adäquat	13<br>Ja
**AF** (Atemfrequenz pro Minute)	29
**RRsys** (systolischer Blutdruck in mmHg)	125
Radialispuls tastbar<br>Rekapillarisierungszeit	Ja<br>2sec
**Gehfähig** (Kann der Patient gehen?)	Nein
**Alter** (Alter des Patienten)	30

Jim, ein Bauarbeiter, war auf dem Public Viewing, um das Spiel mit seinen Freunden zu sehen. Als der Angriff begann, half er sofort dabei, Menschen in Sicherheit zu bringen. Dabei wurde er vom Fahrzeug erfasst und erlitt eine ernste Fraktur am Arm sowie weitere Verletzungen.

Injury Severity (AIS)

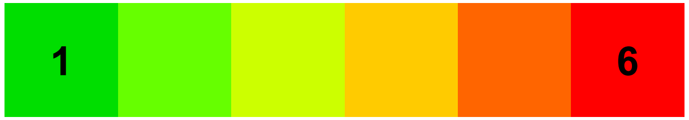

1 gering, 2 moderat, 3 ernst, 4 schwer, 5 kritisch, 6 tödlich

Fraktur Arm	3/6
Fraktur Rippen	2/6
Verstauchung/Zerrung Bein	1/6

Patienten-ID: **041**

Luca Rossi (m)

Luca ist ein Architekt, der aus Italien stammt. Er war in Deutschland, um an einem Bauprojekt teilzunehmen. Beim Public Viewing wollte er sich entspannen, jedoch hat ihn der Vorfall moderat an der Hand verletzt.

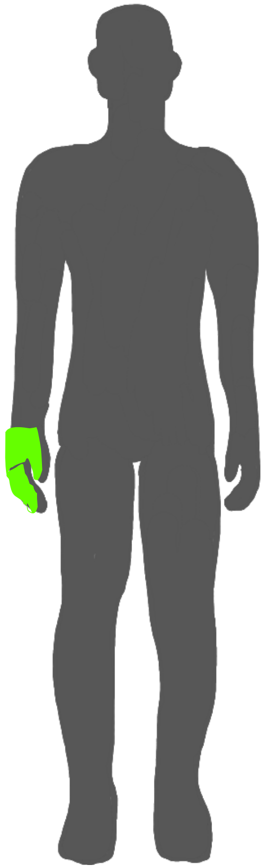

**GCS** (Glasgow-Koma-Skala)<br>Folgt Aufforderungen adäquat	15<br>Ja
**AF** (Atemfrequenz pro Minute)	14
**RRsys** (systolischer Blutdruck in mmHg)	112
Radialispuls tastbar<br>Rekapillarisierungszeit	Ja<br>2sec
**Gehfähig** (Kann der Patient gehen?)	Ja
**Alter** (Alter des Patienten)	51

Dislokation Hand/Finger	2/6
-	-
-	-

Injury Severity (AIS)

1 … **6**

1 gering, 2 moderat, 3 ernst, 4 schwer, 5 kritisch, 6 tödlich

Patienten-ID: **042**

Luca Schuster (m)

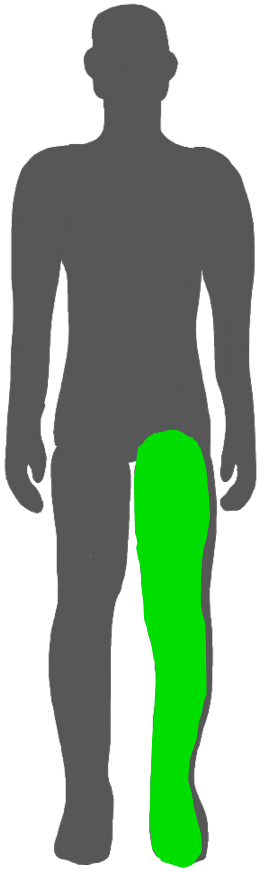

Injury Severity (AIS)

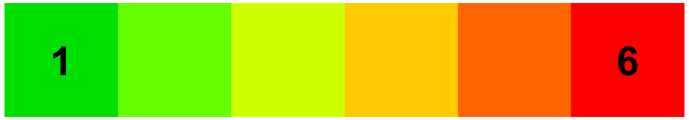

1 gering, 2 moderat, 3 ernst, 4 schwer, 5 kritisch, 6 tödlich

**GCS** (Glasgow-Koma-Skala)<br>Folgt Aufforderungen adäquat	15<br>Ja
**AF** (Atemfrequenz pro Minute)	16
**RRsys** (systolischer Blutdruck in mmHg)	130
Radialispuls tastbar<br>Rekapillarisierungszeit	Ja<br>2sec
**Gehfähig** (Kann der Patient gehen?)	Ja
**Alter** (Alter des Patienten)	43

Verstauchung/Zerrung Bein	1/6
-	-
-	-

Luca, ein IT-Techniker, war auf dem Public Viewing, um kein Spiel zu verpassen. Bei der Flucht vor dem Angreifer stürzte er auf den Rücken und erlitt eine geringe Prellung des rechten Beins. Er ist gehfähig.

Patienten-ID: **043**

Laura Müller (w)

Laura, eine Uni-Mitarbeiterin, war auf dem Public Viewing, um den Abend mit ihren Freunden zu genießen. Als die Tragödie passierte, half sie sofort dabei, Verletzte zu retten und wurde selbst so schwer verletzt, dass sie nicht aufstehen kann.

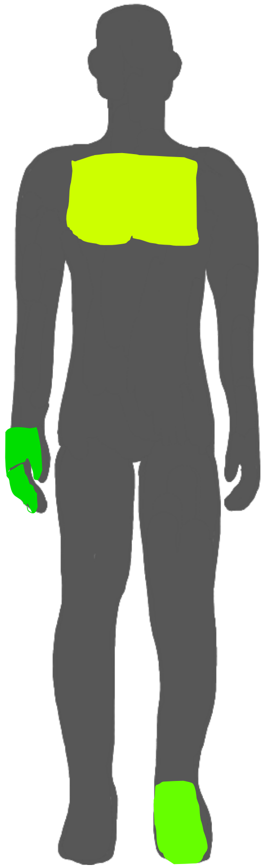

Injury Severity (AIS)

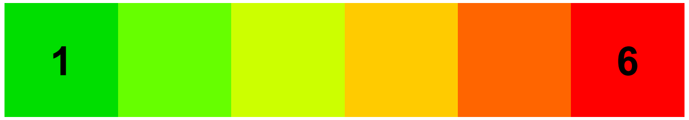

1 gering, 2 moderat, 3 ernst, 4 schwer, 5 kritisch, 6 tödlich

**GCS** (Glasgow-Koma-Skala)<br>Folgt Aufforderungen adäquat	14<br>Ja
**AF** (Atemfrequenz pro Minute)	27
**RRsys** (systolischer Blutdruck in mmHg)	95
Radialispuls tastbar<br>Rekapillarisierungszeit	Ja<br>2sec
**Gehfähig** (Kann der Patient gehen?)	Nein
**Alter** (Alter des Patienten)	61

Quetschung Brust	3/6
Quetschung Fuß	2/6
Quetschung Hand/Finger	1/6

Patienten-ID: **044**

Sophie Wagner (w)

Sophie ist eine pensionierte Lehrerin aus Deutschland. Sie war auf dem Public Viewing, um das Spiel mit ihrer Familie zu genießen. Sie hat Verletzungen an den Armen und am Rücken erlitten und benötigt ärztliche Behandlung, kann aber laufen.

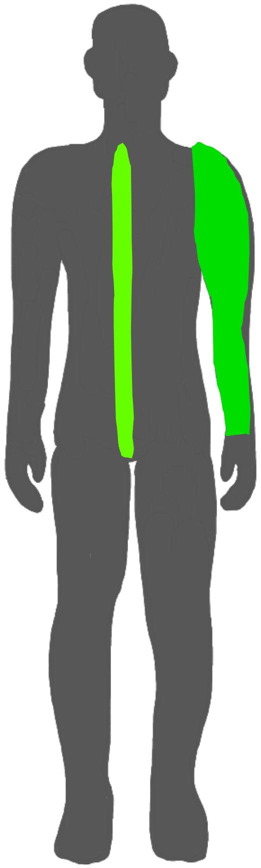

**GCS** (Glasgow-Koma-Skala)<br>Folgt Aufforderungen adäquat	15<br>Ja
**AF** (Atemfrequenz pro Minute)	19
**RRsys** (systolischer Blutdruck in mmHg)	128
Radialispuls tastbar<br>Rekapillarisierungszeit	Ja<br>2sec
**Gehfähig** (Kann der Patient gehen?)	Ja
**Alter** (Alter des Patienten)	66

Verstauchung/Zerrung Arm	1/6
Verstauchung/Zerrung Wirbelsäule	2/6
-	-

Injury Severity (AIS)

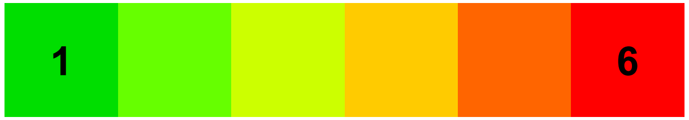

1 gering, 2 moderat, 3 ernst, 4 schwer, 5 kritisch, 6 tödlich

Patienten-ID: **045**

Elena Meier (w)

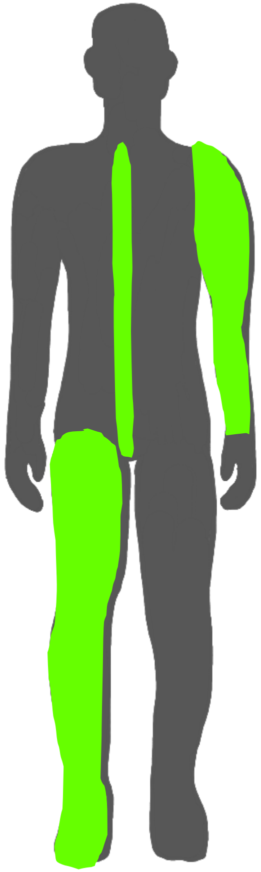

Injury Severity (AIS)

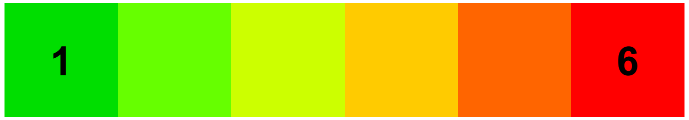

1 gering, 2 moderat, 3 ernst, 4 schwer, 5 kritisch, 6 tödlich

**GCS** (Glasgow-Koma-Skala)<br>Folgt Aufforderungen adäquat	13<br>Ja
**AF** (Atemfrequenz pro Minute)	29
**RRsys** (systolischer Blutdruck in mmHg)	100
Radialispuls tastbar<br>Rekapillarisierungszeit	Ja<br>2sec
**Gehfähig** (Kann der Patient gehen?)	Nein
**Alter** (Alter des Patienten)	46

Dislokation Bein	2/6
Fraktur Arm	2/6
Verstauchung/Zerrung Wirbelsäule	2/6

Elena ist eine russische Geschäftsfrau, die in Deutschland an einer Konferenz teilnimmt. Sie war auf dem Public Viewing, um sich zu entspannen. Das linke Bein ist verdreht, so dass sie mit dem gebrochenen Arm und der Wirbelsäulenprellung nicht aufstehen kann.

Patienten-ID: **046**

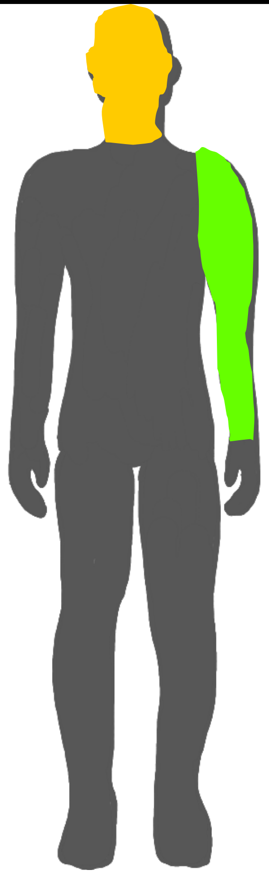

Injury Severity (AIS)

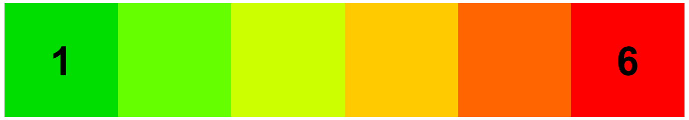

1 gering, 2 moderat, 3 ernst, 4 schwer, 5 kritisch, 6 tödlich

**GCS** (Glasgow-Koma-Skala)<br>Folgt Aufforderungen adäquat	12<br>Nein
**AF** (Atemfrequenz pro Minute)<br>Atemweg frei	25
**RRsys** (systolischer Blutdruck in mmHg)	91
Radialispuls tastbar<br>Rekapillarisierungszeit	Ja<br>2sec
**Gehfähig** (Kann der Patient gehen?)	Nein
**Alter** (Alter des Patienten)	51

Explosionsverletzung Arm	2/6
Explosionsverletzung Kopf	4/6
-	-

Emilia Lehmann (w)

Emilia, eine Lehrerin, genoss den Abend auf dem Public Viewing mit ihren Kollegen. Bei der Explosion war sie nahe am Fahrzeug und erlitt schwere Verletzungen an Kopf und Schädel sowie moderate Verletzungen am rechten Arm.

Patienten-ID: **047**

Lea Fischer (w)

Lea, eine Bankangestellte, war auf dem Public Viewing, um die Nationalmannschaft anzufeuern. Bei der Explosion erlitt Sie durch herumfliegende Metallteile eine ernste Kopfverletzung und eine schwere Quetschverletzung am Bein.

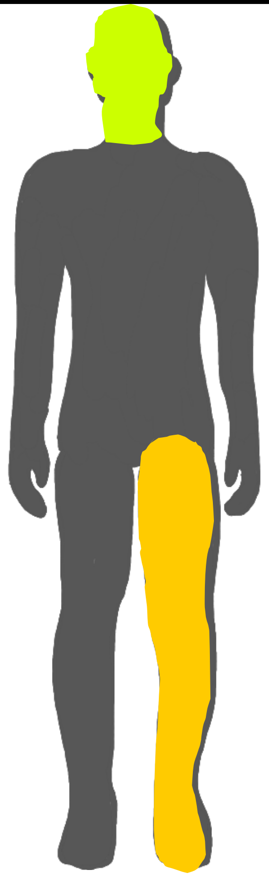

**GCS** (Glasgow-Koma-Skala)<br>Folgt Aufforderungen adäquat	14<br>Ja
**AF** (Atemfrequenz pro Minute)<br>Atemweg frei	22
**RRsys** (systolischer Blutdruck in mmHg)	91
Radialispuls tastbar<br>Rekapillarisierungszeit	Ja<br>2sec
**Gehfähig** (Kann der Patient gehen?)	Nein
**Alter** (Alter des Patienten)	38

Explosionsverletzung Kopf	3/6
Quetschung Bein	4/6
-	-

Injury Severity (AIS)

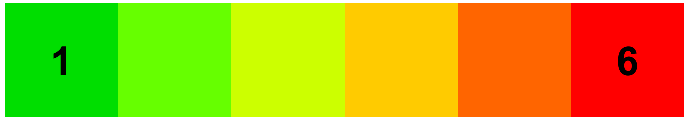

1 gering, 2 moderat, 3 ernst, 4 schwer, 5 kritisch, 6 tödlich

Patienten-ID: **048**

Noah Zimmermann (m)

Noah, ein Dozent, war auf dem Public Viewing, um das Spiel mit seinen Freunden zu sehen. Beim Sturz in die Tiefe landete er so ungeschickt, dass beide Beine so verdreht sind, dass er nicht auftreten kann.

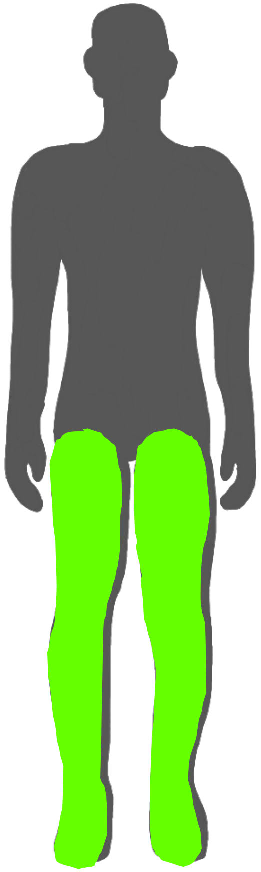

**GCS** (Glasgow-Koma-Skala)<br>Folgt Aufforderungen adäquat	15<br>Ja
**AF** (Atemfrequenz pro Minute)	18
**RRsys** (systolischer Blutdruck in mmHg)	167
Radialispuls tastbar<br>Rekapillarisierungszeit	Ja<br>2sec
**Gehfähig** (Kann der Patient gehen?)	Nein
**Alter** (Alter des Patienten)	51

Dislokation Bein	2/6
Dislokation Bein	2/6
-	-

Injury Severity (AIS)

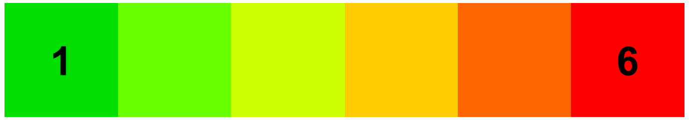

1 gering, 2 moderat, 3 ernst, 4 schwer, 5 kritisch, 6 tödlich

Patienten-ID: **049**

Lukas Hoffmann (m)

Lukas, ein Fußballspieler war auf dem Public Viewing, um das Spiel mit seinen Freunden zu erleben. Als der Angriff geschah, erlitt er zahlreiche Verletzungen. Vor allem das Abdomen ist sehr schmerzhaft.

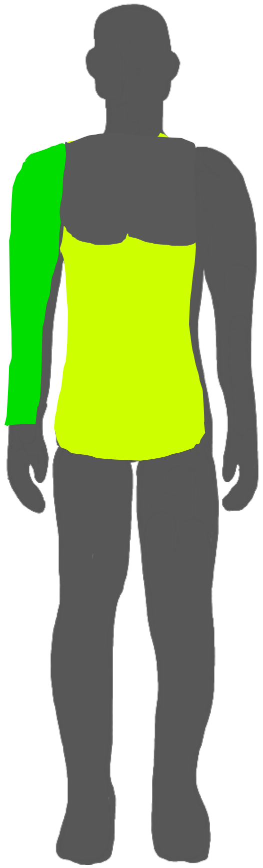

Injury Severity (AIS)

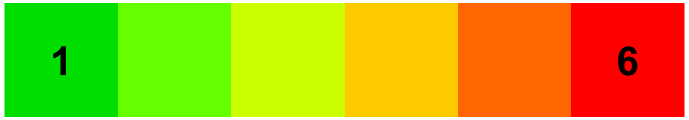

1 gering, 2 moderat, 3 ernst, 4 schwer, 5 kritisch, 6 tödlich

**GCS** (Glasgow-Koma-Skala)<br>Folgt Aufforderungen adäquat	12<br>Nein
**AF** (Atemfrequenz pro Minute)	34
**RRsys** (systolischer Blutdruck in mmHg)	65
Radialispuls tastbar<br>Rekapillarisierungszeit	Nein<br>4sec
**Gehfähig** (Kann der Patient gehen?)	Nein
**Alter** (Alter des Patienten)	44

Amputation Hand/Finger<br>Blutung steht	2/6
Quetschung Abdomen	3/6
-	-

Patienten-ID: **050**

David Smith (m)

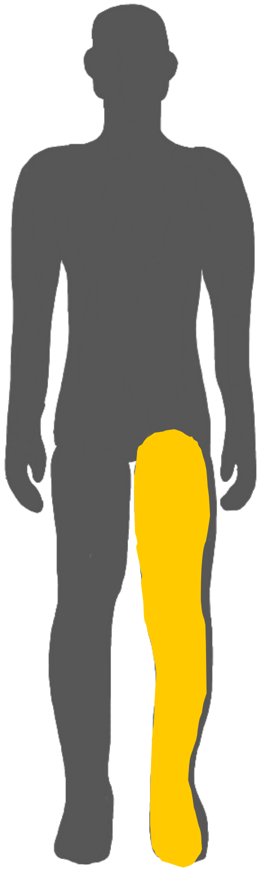

Injury Severity (AIS)

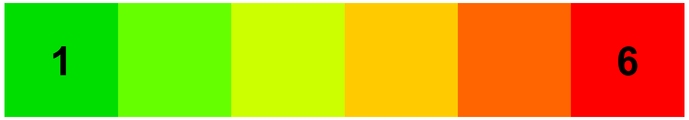

1 gering, 2 moderat, 3 ernst, 4 schwer, 5 kritisch, 6 tödlich

**GCS** (Glasgow-Koma-Skala)<br>Folgt Aufforderungen adäquat	13<br>Ja
**AF** (Atemfrequenz pro Minute)	28
**RRsys** (systolischer Blutdruck in mmHg)	93
Radialispuls tastbar<br>Rekapillarisierungszeit	Ja<br>2sec
**Gehfähig** (Kann der Patient gehen?)	Nein
**Alter** (Alter des Patienten)	27

Quetschung Bein	4/6
-	-
-	-

David ist ein Tourist aus den USA, der Deutschland besucht. Er war auf dem Public Viewing, um die lokale Kultur kennenzulernen. Er hat sich eine schwere Quetschverletzung am Oberschenkel zugezogen und ist nicht gehfähig.

Patienten-ID: **051**

Mia Klein (w)

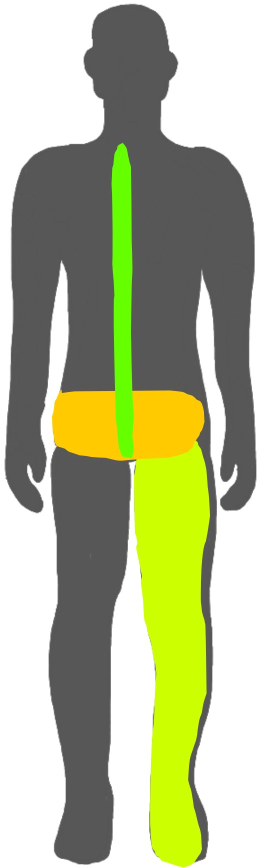

Injury Severity (AIS)

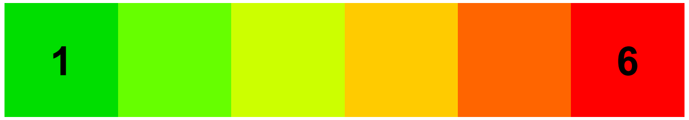

1 gering, 2 moderat, 3 ernst, 4 schwer, 5 kritisch, 6 tödlich

**GCS** (Glasgow-Koma-Skala)<br>Folgt Aufforderungen adäquat	14<br>Ja
**AF** (Atemfrequenz pro Minute)	33
**RRsys** (systolischer Blutdruck in mmHg)	91
Radialispuls tastbar<br>Rekapillarisierungszeit	Ja<br>2sec
**Gehfähig** (Kann der Patient gehen?)	Nein
**Alter** (Alter des Patienten)	42

Fraktur Bein	3/6
Fraktur Becken/Beckenring	4/6
Verstauchung/Zerrung Wirbelsäule	2/6

Mia, eine Büroleiterin, genoss den Abend auf dem Public Viewing mit ihren Freunden. Von der Explosion weggeschleudert brach sie sich das rechte Bein, das deutlich Umfangsvergrößert am Oberschenkel ist.

Patienten-ID: **052**

Marie Berger (w)

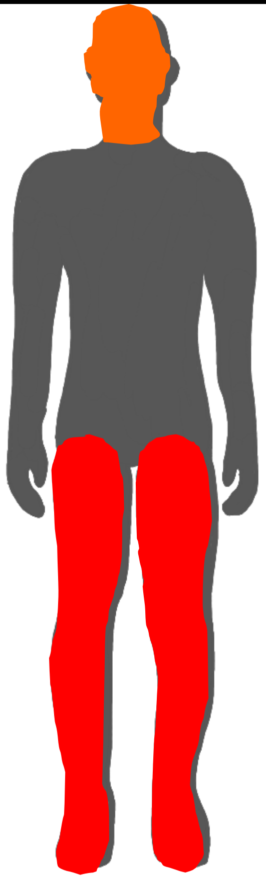

Injury Severity (AIS)

1					6

1 gering, 2 moderat, 3 ernst, 4 schwer, 5 kritisch, 6 tödlich

**GCS** (Glasgow-Koma-Skala)<br>Folgt Aufforderungen adäquat	3<br>Nein
**AF** (Atemfrequenz pro Minute)	0
**RRsys** (systolischer Blutdruck in mmHg)	13
Radialispuls tastbar<br>Rekapillarisierungszeit	Nein<br>-
**Gehfähig** (Kann der Patient gehen?)	Nein
**Alter** (Alter des Patienten)	44

Amputation beider Beine	6/6
Quetschung Kopf	5/6
-	-

Marie, eine angestellte Physiotherapeutin, wollte mit ihren Kolleginnen das Public Viewing genießen. Sie wurde durch die Explosion lebensgefährlich verletzt: Beide Beine wurden auf Höhe der Leiste komplett abgetrennt

Patienten-ID: **053**

Matteo Russo (m)

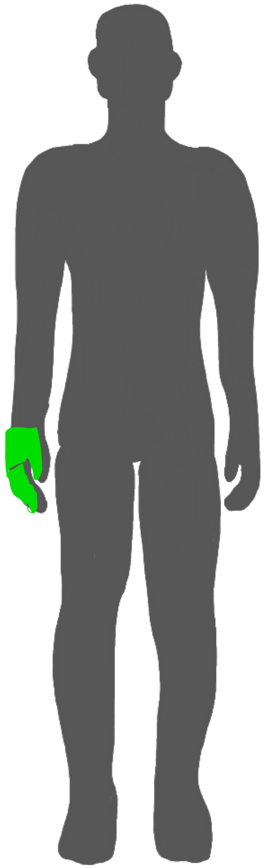

**GCS** (Glasgow-Koma-Skala)<br>Folgt Aufforderungen adäquat	15<br>Ja
**AF** (Atemfrequenz pro Minute)	17
**RRsys** (systolischer Blutdruck in mmHg)	144
Radialispuls tastbar<br>Rekapillarisierungszeit	Ja<br>2sec
**Gehfähig** (Kann der Patient gehen?)	Ja
**Alter** (Alter des Patienten)	58

Dislokation Hand/Finger	1/6
-	-
-	-

Matteo ist ein pensionierter italienischer Polizeibeamter, der Deutschland besucht hat. Er war auf dem Public Viewing, um die Atmosphäre zu genießen. Die plötzliche Tragödie hat ihn geschockt und er unterstützt trotz Verletzung die Rettungskräfte so gut er kann.

Injury Severity (AIS)

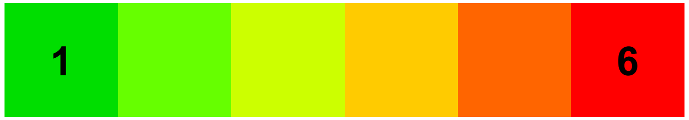

1 gering, 2 moderat, 3 ernst, 4 schwer, 5 kritisch, 6 tödlich

Patienten-ID: **054**

Anna Müller (w)

Anna ist eine deutsche Studentin und großer Fußballfan. Beim ersten Spiel wollte sie unbedingt dabei sein. Sie hat sich leichte Verletzungen am rechten Arm und eine Rippenfraktur zugezogen, kümmert sich jetzt um ihre Freunde.

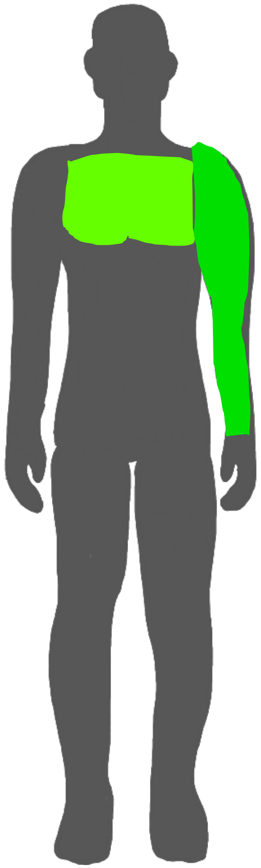

Injury Severity (AIS)

1					6

1 gering, 2 moderat, 3 ernst, 4 schwer, 5 kritisch, 6 tödlich

**GCS** (Glasgow-Koma-Skala)<br>Folgt Aufforderungen adäquat	15<br>Ja
**AF** (Atemfrequenz pro Minute)	18
**RRsys** (systolischer Blutdruck in mmHg)	169
Radialispuls tastbar<br>Rekapillarisierungszeit	Ja<br>2sec
**Gehfähig** (Kann der Patient gehen?)	Ja
**Alter** (Alter des Patienten)	23

Quetschung Arm	1/6
Fraktur Rippen	2/6
-	-

Patienten-ID: **055**

Ali Al-Mansouri (m)

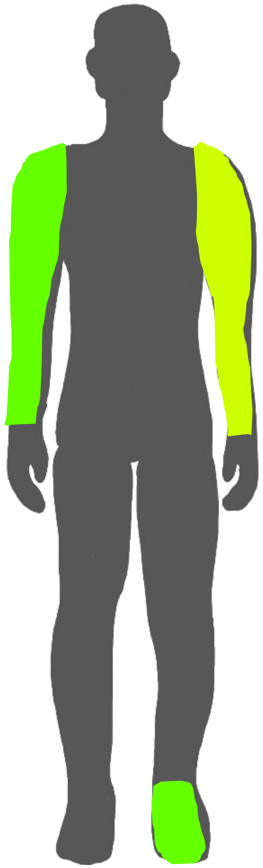

Injury Severity (AIS)

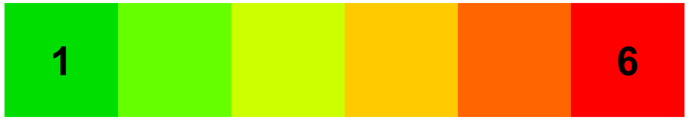

1 gering, 2 moderat, 3 ernst, 4 schwer, 5 kritisch, 6 tödlich

**GCS** (Glasgow-Koma-Skala)<br>Folgt Aufforderungen adäquat	14<br>Ja
**AF** (Atemfrequenz pro Minute)	29
**RRsys** (systolischer Blutdruck in mmHg)	91
Radialispuls tastbar<br>Rekapillarisierungszeit	Ja<br>2sec
**Gehfähig** (Kann der Patient gehen?)	Nein
**Alter** (Alter des Patienten)	40

Explosionsverletzung Arm	3/6
Quetschung Fuß	2/6
Fraktur Arm	2/6

Ali ist ein Geschäftsmann aus dem Nahen Osten, der an einer Konferenz in Deutschland teilgenommen hat. Er war auf dem Public Viewing, um sich zu entspannen und wurde durch die Explosion an Armen und Fuß verletzt. Er ist nicht gehfähig.

Patienten-ID: **056**

Marco Ferrari (m)

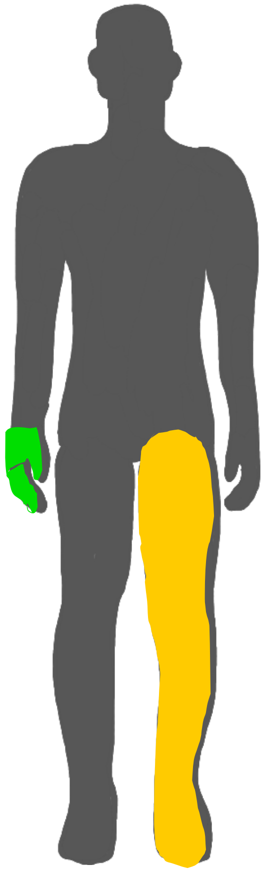

**GCS** (Glasgow-Koma-Skala)<br>Folgt Aufforderungen adäquat	13<br>Ja
**AF** (Atemfrequenz pro Minute)	29
**RRsys** (systolischer Blutdruck in mmHg)	89
Radialispuls tastbar<br>Rekapillarisierungszeit	Ja<br>2sec
**Gehfähig** (Kann der Patient gehen?)	Nein
**Alter** (Alter des Patienten)	19

Quetschung Bein	4/6
Verstauchung/Zerrung Hand/Finger	1/6
-	-

Marco ist ein Student aus Italien, der ein Auslandssemester in Deutschland macht. Er war auf dem Public Viewing, um sich von seinem Studium zu entspannen. Auf der Flucht ist er im Gemenge gestürzt, hat sich eine schwere Quetschverletzung am Oberschenkel zugezogen und kann nicht laufen.

Injury Severity (AIS)

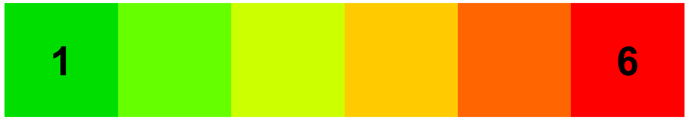

1 gering, 2 moderat, 3 ernst, 4 schwer, 5 kritisch, 6 tödlich

Patienten-ID: **057**

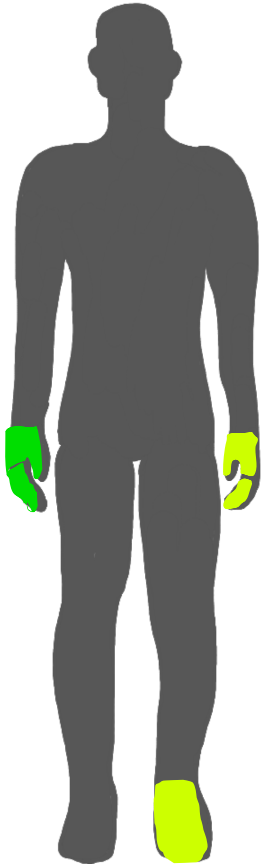

Injury Severity (AIS)

1					6

1 gering, 2 moderat, 3 ernst, 4 schwer, 5 kritisch, 6 tödlich

**GCS** (Glasgow-Koma-Skala)<br>Folgt Aufforderungen adäquat	15<br>Ja
**AF** (Atemfrequenz pro Minute)	17
**RRsys** (systolischer Blutdruck in mmHg)	139
Radialispuls tastbar<br>Rekapillarisierungszeit	Ja<br>2sec
**Gehfähig** (Kann der Patient gehen?)	Nein
**Alter** (Alter des Patienten)	29

Amputation Hand/Finger<br>Blutung steht	3/6
Fraktur Fuß	3/6
Zerrung/Verstauchung Hand Finger	1/6

Sebastian Becker (m)

Sebastian, ein Verkäufer, befand sich mit seiner Familie auf dem Public Viewing, um das Spiel zu erleben. Als der Angriff geschah, wollte er alle in Sicherheit bringen, blieb dabei an einem Zaun hängen und amputierte sich dabei einen Finger, brach sich den rechten Fuß und prellte sich die linke Hand. Er ist so nicht mehr gehfähig.

Patienten-ID: **058**

Sophia Weber (w)

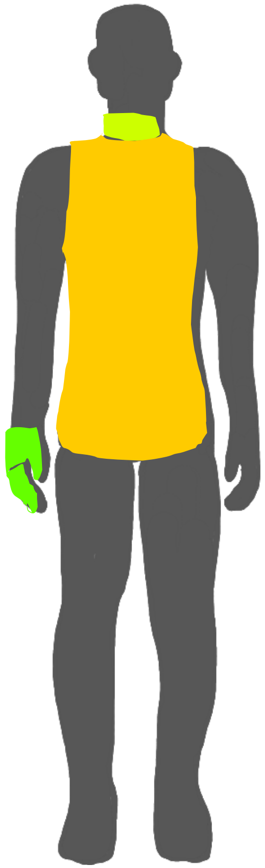

Injury Severity (AIS)

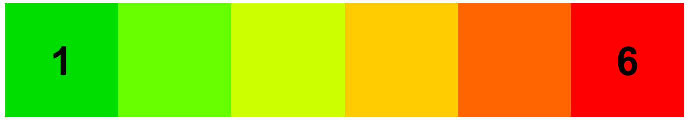

1 gering, 2 moderat, 3 ernst, 4 schwer, 5 kritisch, 6 tödlich

**GCS** (Glasgow-Koma-Skala)<br>Folgt Aufforderungen adäquat	13<br>Ja
**AF** (Atemfrequenz pro Minute)	34
**RRsys** (systolischer Blutdruck in mmHg)	93
Radialispuls tastbar<br>Rekapillarisierungszeit	Ja<br>2sec
**Gehfähig** (Kann der Patient gehen?)	Nein
**Alter** (Alter des Patienten)	25

Dislokation Hand/Finger	2/6
Fraktur Brust	4/6
Verstauchung/Zerrung Nacken	3/6

Sophia, eine junge Anwältin, genoss den Abend auf dem Public Viewing mit ihren Freunden. Beim Kontakt mit dem Fahrzeug des Angreifers zog sie sich eine schwere Rippenfraktur zu.

Patienten-ID: **059**

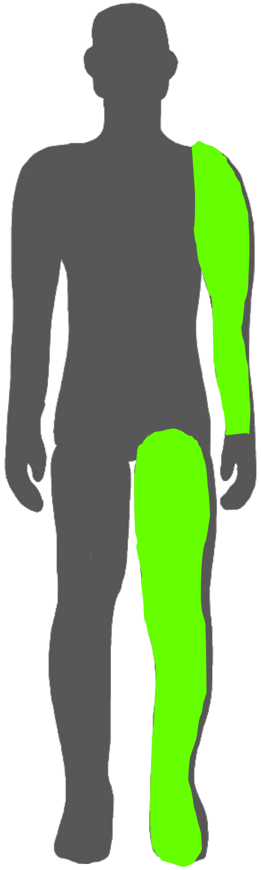

Injury Severity (AIS)

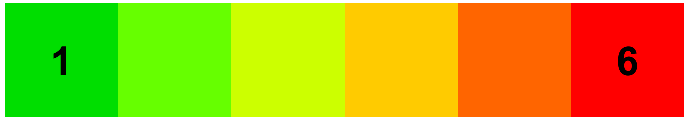

1 gering, 2 moderat, 3 ernst, 4 schwer, 5 kritisch, 6 tödlich

**GCS** (Glasgow-Koma-Skala)<br>Folgt Aufforderungen adäquat	13<br>Ja
**AF** (Atemfrequenz pro Minute)	24
**RRsys** (systolischer Blutdruck in mmHg)	100
Radialispuls tastbar<br>Rekapillarisierungszeit	Ja<br>2sec
**Gehfähig** (Kann der Patient gehen?)	Nein
**Alter** (Alter des Patienten)	34

Dislokation Bein	2/6
Fraktur Arm	2/6
-	

Julia Müller (w)

Julia ist eine deutsche Lehrerin, die gerne Fußballspiele schaut. Sie war mit ihren Freunden auf dem Public Viewing und hat Verletzungen an den Beinen und Armen erlitten und kann nicht aufstehen.

Patienten-ID: **060**
