# Supplementary material for: Evaluation of an App-Based Mobile Triage System for Mass Casualty Incidents: Within-Subjects Experimental Study
Source: J Med Internet Res. 2024 Nov 21;26:e65728. doi: 10.2196/65728 (PMC11621716; doi:10.2196/65728)
Supplement: Multimedia Appendix 4 [file jmir_v26i1e65728_app4.pdf]

Anhängekarte für Verletzte/Kranke

Nur für Übungszwecke

Registration card for injured/sick persons – Fiche d´enregistrement pour blessés/malades

**Name**<br>Name<br>Nom	**Patienten-Nr.** aufkleben
**Vorname**<br>First name<br>Prénom	
**Geburtsdatum/~Alter**<br>Date of birth/~age<br>Date de naissance/~âge	♂ m / ♀ f / ⚧ d
**Nationalität**<br>Nationality<br>Nationalité	**Datum**<br>Date

**Sichtung**<br>Sorting/Triage<br>**Kategorie**<br>Category<br>Catégorie	**1. Sichtung**<br>**Uhrzeit/Name**<br>Time/Name<br>Heure/Nom	**2. Sichtung**<br>**Uhrzeit/Name**	**3. Sichtung**<br>**Uhrzeit/Name**	**4. Sichtung**<br>**Uhrzeit/Name**
**I**				
**II**				
**III**				
**IV**				

**Transportmittel**<br>Transportation<br>Moyen de transport	**Transportziel**<br>Destination

**Transport**<br>Transportation<br>Transport	**liegend**<br>lying<br>couche	**sitzend**<br>sitting<br>assis	**mit Notarzt**<br>with doctor<br>avec médecin	**isoliert**<br>isolated<br>isolé	**Priorität**<br>Priority<br>Priorité
			☺	( )	a ○<br>b ○

**Innenliegende Suchdienstkarte** enclosed card for tracing service, fiche d'enregistrement ci-jointe		
**1. Ausfertigung**<br>1st Copy, 1ère Copie	**weitergeleitet**<br>referred, acheminé	○
**2. Ausfertigung**<br>2nd Copy, 2ème Copie	**weitergeleitet**<br>referred, acheminé	○

© Deutsches Rotes Kreuz Generalsekretariat 09/2021

Kurz-Diagnose
short diagnose
diagnostic bref

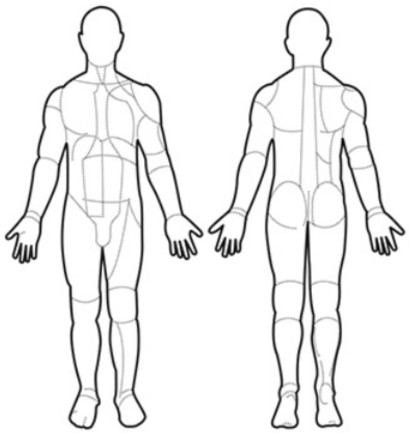

**Verletzung**<br>injury<br>blessure	
**Verbrennung**<br>burn<br>brûlure	
**Erkrankung**<br>disease<br>maladie	
**Vergiftung**<br>intoxication	
**Verstrahlung**<br>excessive radiation<br>radiation excessive	
**Psyche**<br>psychic condition<br>état psychique	

**Zustand/Uhrzeit**<br>state/time<br>état/heure		
**Bewusstsein**<br>consciousness<br>connaissance	o.B.	○
	↓	○
**Atmung**<br>respiration	o.B.	○
	↓	○
**Kreislauf**<br>circulation	o.B.	○
	↓	○

**Erst-Therapie**<br>first therapy<br>thérapie première	
**Infusion**<br>infusion	○
**Analgetika**<br>analgesics<br>analgésique	○
**Antidote**<br>antidots<br>antidote	○
**sonstige Medikamente**<br>other drugs<br>autres médicaments	○

Bemerkungen
notes
remarques

© Deutsches Rotes Kreuz Generalsekretariat 09/2021

Nur für Übungszwecke
