# Supplementary material for: Evaluation of an App-Based Mobile Triage System for Mass Casualty Incidents: Within-Subjects Experimental Study
Source: J Med Internet Res. 2024 Nov 21;26:e65728. doi: 10.2196/65728 (PMC11621716; doi:10.2196/65728)
Supplement: Multimedia Appendix 5 [file jmir_v26i1e65728_app5.pdf]

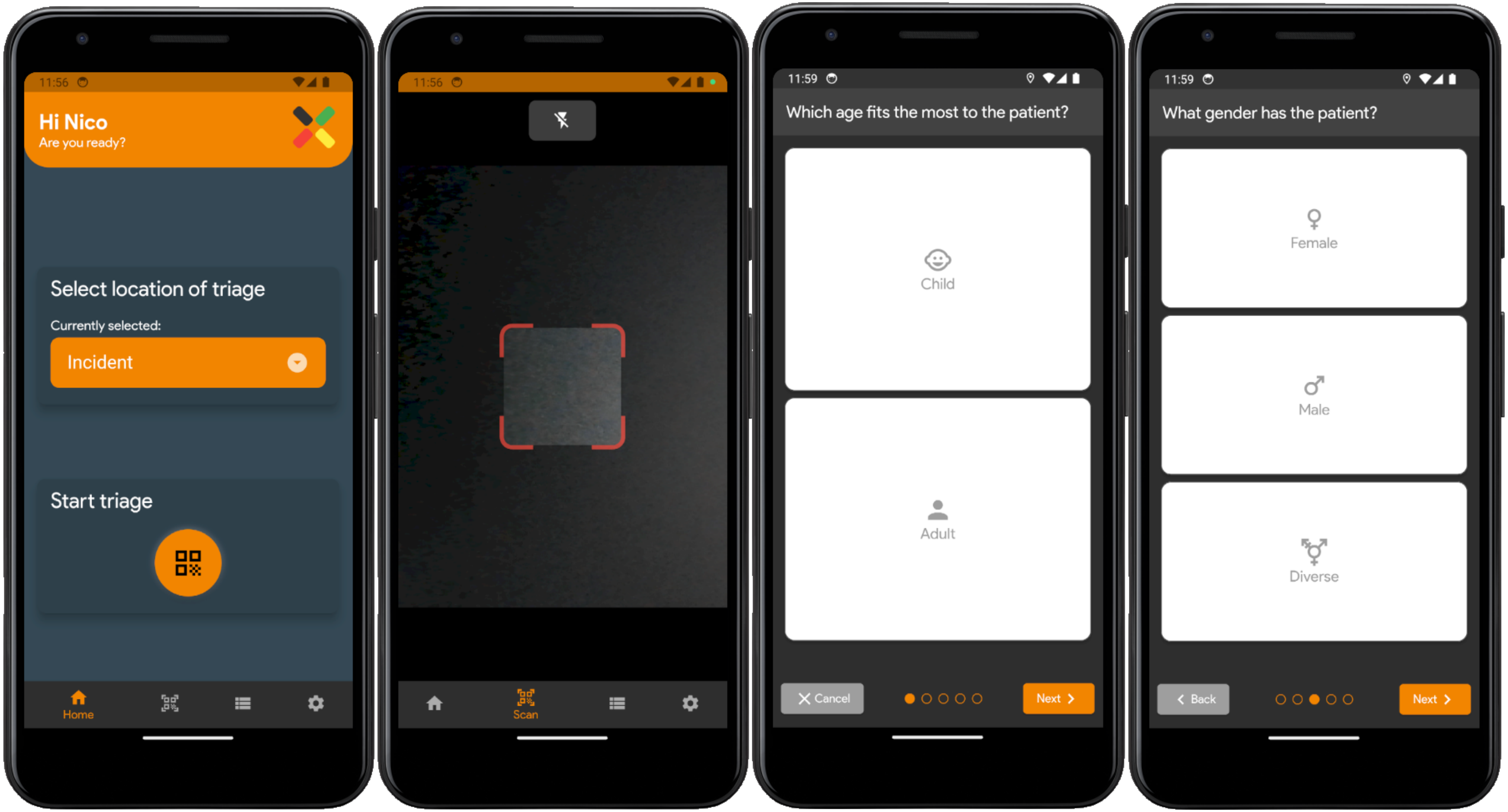
11:56
Hi Nico
Are you ready?
Select location of triage
Currently selected:
Incident
Start triage
Home
11:56
Scan
11:59
Which age fits the most to the patient?
Child
Adult
Cancel
Next
11:59
What gender has the patient?
Female
Male
Diverse
Back
Next

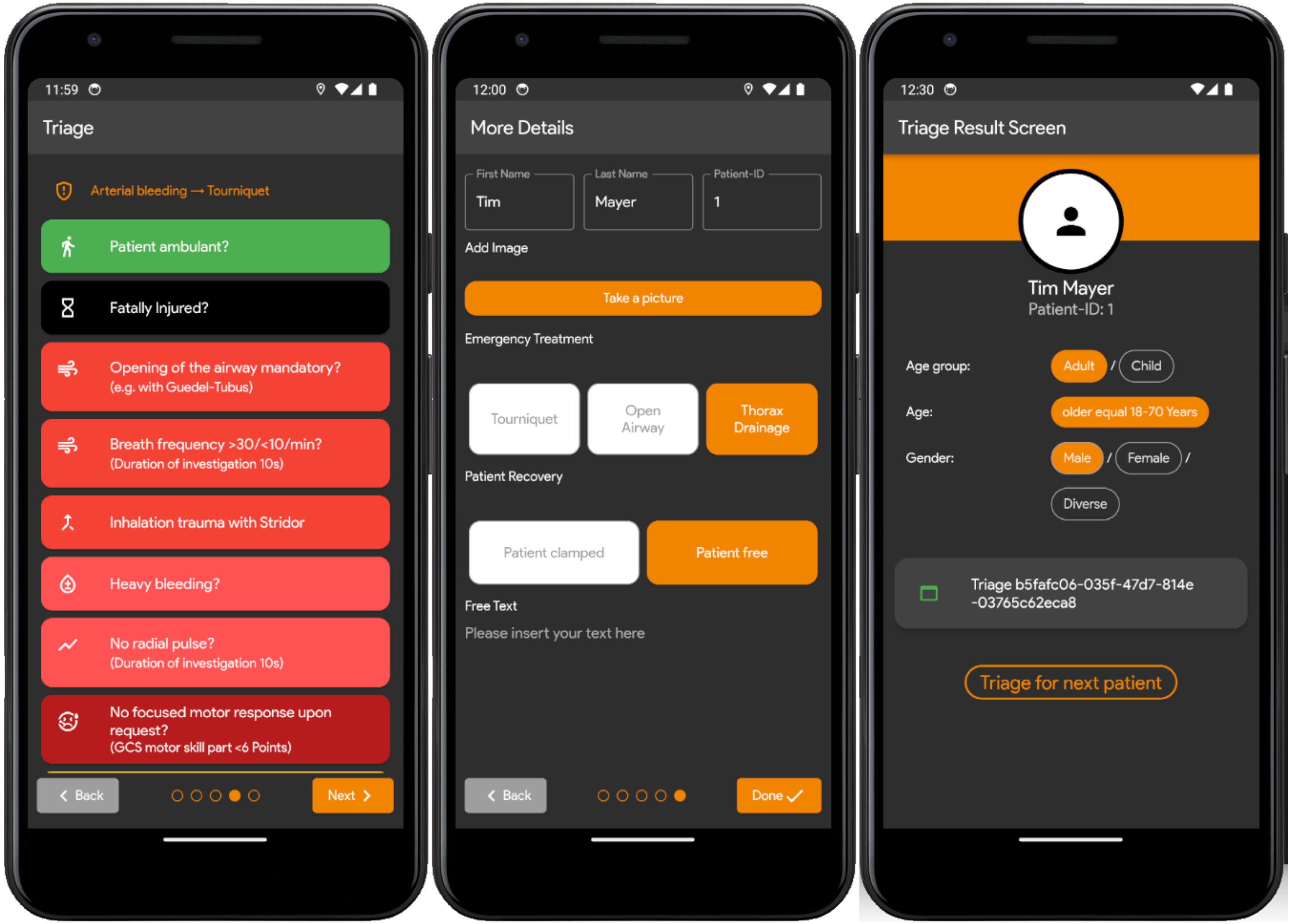
11:59
Triage
Arterial bleeding → Tourniquet
Patient ambulant?
Fatally Injured?
Opening of the airway mandatory?
(e.g. with Guedel-Tubus)
Breath frequency >30/<10/min?
(Duration of investigation 10s)
Inhalation trauma with Stridor
Heavy bleeding?
No radial pulse?
(Duration of investigation 10s)
No focused motor response upon request?
(GCS motor skill part <6 Points)
Back
Next
12:00
More Details
First Name
Tim
Last Name
Mayer
Patient-ID
1
Add Image
Take a picture
Emergency Treatment
Tourniquet
Open Airway
Thorax Drainage
Patient Recovery
Patient clamped
Patient free
Free Text
Please insert your text here
Back
Done
12:30
Triage Result Screen
Tim Mayer
Patient-ID: 1
Age group:
Adult / Child
Age:
older equal 18-70 Years
Gender:
Male / Female /
Diverse
Triage b5fafc06-035f-47d7-814e-03765c62eca8
Triage for next patient
