# Supplementary material for: Evaluation of an App-Based Mobile Triage System for Mass Casualty Incidents: Within-Subjects Experimental Study
Source: J Med Internet Res. 2024 Nov 21;26:e65728. doi: 10.2196/65728 (PMC11621716; doi:10.2196/65728)
Supplement: Multimedia Appendix 6 [file jmir_v26i1e65728_app6.docx]

**German Version of the User Experience Questionnaire as used in our study with English translations.**

Please see https://www.ueq-online.org for further information and references.

1. annoying – enjoyable (unerfreulich – erfreulich; A)

2. not understandable – understandable (unverständlich – verständlich; P)

3. creative – dull (kreativ – phantasielos; N)

4. easy to learn – difficult to learn (leicht zu lernen – schwer zu lernen, P)

5. valuable – inferior (wertvoll – minderwertig; S)

6. boring – exiting (langweilig – spannend; S)

7. not interesting – interesting (uninteressant – interessant; S)

8. unpredictable – predictable (unberechenbar – voraussagbar; D)

9. fast – slow (schnell – langsam; E)

10. inventive – conventional (originell – konventionell; N)

11. obstructive – supportive (behindernd – unterstützend; D)

12. good – bad (gut – schlecht; A)

13. complicated – easy (kompliziert – einfach; P)

14. unlikable – pleasing (abstoßend – anziehend; A)

15. usual – leading edge (herkömmlich – neuartig; N)

16. unpleasant – pleasant (Uaangenehm – angenehm; A)

17. secure – not secure (sicher – unsicher; D)

18. motivating – demotivating (aktivierend – einschläfernd; S)

19. meets expectations – does not meet expectations (erwartungskonform – nicht

erwartungskonform; D)

20. inefficient – efficient (ineffizient – effizient; E)

21. clear – confusing (uebersichtlich – verwirrend; P)

22. impractical – practical (unpragmatisch – pragmatisch; E)

23. organized – cluttered (aufgeräumt – überladen; E)

24. attractive – unattractive (attraktiv – unattraktiv; A)

25. friendly – unfriendly (sympatisch – unsympathisch; A)

26. conservative – innovative (lonservativ – innovative; N)

A = Attractiveness

P = Perspicuity

N = Novelty

S = Stimulation

D = Dependability

E = Efficiency
