# Supplementary material for: Evaluation of an App-Based Mobile Triage System for Mass Casualty Incidents: Within-Subjects Experimental Study
Source: J Med Internet Res. 2024 Nov 21;26:e65728. doi: 10.2196/65728 (PMC11621716; doi:10.2196/65728)
Supplement: Multimedia Appendix 7 [file jmir_v26i1e65728_app7.pdf]

Kendall tau correlations between age and the outcome variables to check the conditions for a multivariate analysis of covariance.

Outcome variable	Tool	Kendall tau	*P* value
**Triage accuracy**	Paper-based	.181	.26
	KatApp	-.229	.09
**Duration (minutes)**	Paper-based	-.205	.07
	KatApp	.082	.47
**Subjective Rating**	Paper-based	.167	.19
	KatApp	-.069	.62
**User Experience Questionnaire**			
Attractiveness	Paper-based	.043	.71
	KatApp	.006	.96
Efficiency	Paper-based	.100	.397
	KatApp	.021	.86
Perspicuity	Paper-based	.001	.99
	KatApp	.046	.70
Dependability	Paper-based	.012	.92
	KatApp	.131	.28
Stimulation	Paper-based	-.037	.75
	KatApp	.124	.29
Novelty	Paper-based	-.037	.76
	KatApp	-.088	.36
