# Supplementary material for: Evaluation of an App-Based Mobile Triage System for Mass Casualty Incidents: Within-Subjects Experimental Study
Source: J Med Internet Res. 2024 Nov 21;26:e65728. doi: 10.2196/65728 (PMC11621716; doi:10.2196/65728)
Supplement: Multimedia Appendix 8 [file jmir_v26i1e65728_app8.pdf]

TIDieR

App-based Mobile Triage System for Mass Casualty Incidents: Within-Subjects Experimental Study

Why: An app-based triage tool for emergency and disaster medicine may be superior to paper-based triage tools in terms of speed, accuracy and user experience, and therefore address weaknesses and problems of the current paper-based triage process by reducing time-consuming and error-prone manual tasks. This could further expand the potential of digitalization to optimize processes in disaster medicine, which in turn could save more lives.

What (material): The study aimed to evaluate the KatApp as a triage tool for emergency and disaster medicine which was previously designed and developed within our research group as described in the background. The objective was to determine if the KatApp is superior to paper-based triage tools in terms of speed, accuracy, and user experience among emergency medicine experts. We hypothesized that the KatApp would be at least as accurate as the paper-based tool, but faster and more user-friendly.

TRIAGE TOOLS

Paper-based Tool: When using the conventional paper-based tool, participants had to fill out a casualty card (*Multimedia Appendix 4*) from their equipment that also required them to fold the triage category manually and attach the card to the patient card. In addition, the participants had to record the triage category on a documentation sheet. To facilitate the triage, the participants were given an mSTART checklist.

KatApp: When using the KatApp, participants had to attach a QR code from their equipment to each patient, scan it with the KatApp QR code scanner and conduct the triage process as described above (*Figure 1*) and displayed in *Multimedia Appendix 5*. Participants used their mobile smartphones which were connected to the mobile network. The triage results were immediately forwarded to a central dashboard.

All software components are managed using repositories on GitHub (2024 GitHub, Inc). Automatic deployments are implemented using GitHub Actions and custom scripts. The iOS app is deployed to TestFlight from Apple, while the Android app is deployed to Firebase. Rescue teams can install the desired version from these platforms in advance of a training. The dashboard web application and the back-end are both deployed to Amazon Web Services AWS (2023 Amazon Web Services, Inc.). The dashboard is hosted as a web application on AWS, while the back-end services are implemented as AWS Lambda functions. These services use AWS DynamoDB for user management, logging, and triage records, and AWS S3 for binary data. *Figure 3* depicts the architecture in more detail.

The KatApp system is a distributed system that relies on the connectivity of its nodes, including the mobile devices of rescue teams, the tablets of the command post, and the back-end services. The standard operation mode assumes an internet connection is available around the incident scene, allowing the web application and back-end services deployed in the cloud to be accessed by the apps on the rescue teams' mobile devices. The dashboard web application is reachable via browsers on the command post's tablets or computers and can establish a web socket connection to the KatApp back-end. In a perfect world, this operation mode would be sufficient. As we know from the fallacies of distributed computing, networks are not reliable in many respects, especially during disasters when critical communication infrastructure might be destroyed or overstressed, or in regions with poor network coverage. To address this, the KatApp system implements a caching mechanism in the triage app, allowing rescue teams to continue triaging patients even without an internet connection. The triage records are stored locally and transmitted automatically once the connection is restored. Additionally, the KatApp system supports an operation mode independent of an internet connection. A local wireless network can be set up by the command post or a technical organization on-site, with a server containing the dashboard application and back-end services. Mobile devices connect to this network, allowing the system to function as usual. Since the back-end services were developed as AWS Lambda functions, they cannot run locally. Therefore, an additional abstraction layer was implemented to use alternative open-source or self-written components for each AWS component.

More information: https://docs.katapp.org

PATIENT CARDS

The patient cards were initially designed using a previously designed artificial patient database and then further adapted by the study team. They contained a pictogram to represent the injury pattern and information on the injury severity (Abbreviated Injury Scale, AIS), vital parameters (i.e. Glasgow Coma Scale [GCS], respiratory rate, systolic blood pressure, radial pulse, re-capillarization time, walking ability, age), as well as a short storyboard with additional information on the course of the accident (*Multimedia Appendix 3*). In order to keep the two sessions comparable, the number of patient cards per triage category was identical (red: 20% [6/30], yellow: 43.3% [13/30], green: 30% [9/30], black: 6.7% [2/30]), while the categorization was based on empirical data on mass casualty incidents. In addition, we matched pairs of patient cards in terms of overall injury severity (AIS) and injury pattern to minimize the impact of differences in patient cards on outcome variables between the triage tools. The order of patient cards in each course was randomized using the online randomization tool RANDOM.ORG (Randomness and Integrity Services Limited, Dublin, 2024), but equal for each participant.

OUTCOME MEASURES

Duration and Quality of Triages: The study staff recorded the time at the beginning and end of each parcours as soon as the participants crossed the start and finish lines. This timing included the entire triage process: walking between patients, applying QR codes or attaching paper triage cards (depending on the method used), and conducting the triage assessment itself. In addition, the number of correct triages per session was assessed by analyzing participants’ documentation sheets of the paper-based tool, and the data that was collected by the KatApp.

Subjective Ratings: The participants had to rate both triage tools as a school grade of the German grading system from 1 (excellent) to 6 (insufficient). They were also asked about their preference with a forced-choice item.

User Experience Questionnaire: We used the German version of the User Experience Questionnaire (UEQ) to assess the participants' experience after each triage tool. The UEQ consists of 26 items that are grouped into six scales (Attractiveness, Perspicuity, Efficiency, Dependability, Stimulation, Novelty), while each item displays a semantic differential that must be rated on a 7-point Likert scale, which are in turn coded from -3 to +3 (*Multimedia Appendix 6*). *Attractiveness* refers to the overall impression of the product (Do user like or dislike the product?), whereas *Perspicuity* assesses the ease to get familiar with the product and to learn how to use it. In addition, *Efficiency* displays whether users can solve their tasks without unnecessary efforts, and *Dependability* refers to the feeling of control of the interaction, security and predictability. *Stimulation* measures how exciting and motivating it is to use the product, and *Novelty* presents whether the product is creative and catches the interest of users. *Figure 4* illustrates the overall experimental design.

URL: https://www.ueq-online.org/

What (procedures): The study was conceived as a within-subjects experimental design, with the triage tool as within-subjects factor (paper-based tool vs. KatApp). The order of the triage tools was counterbalanced to compensate for carry-over effects. The study was conducted on the premises of the German Red Cross in Kirchentellinsfurt on behalf of the University Hospital Tübingen together with Reutlingen University in June 2024. The study participation was voluntary. After participants registered for the triage training course and study, they were provided with instructions on how to install the app and information regarding the study procedure one week prior to the course.
To familiarize the participants with the mSTART algorithm using both the paper-based tool as well as the KatApp, they were given an introduction at the beginning of the course. They were then asked to complete two triage sessions in the context of a simulated mass casualty incident, which would be caused by a terrorist driving into the Fan Zone of a public screening of the UEFA European Football Championship 2024. In one session, they were using the KatApp, and in the respective other session the conventional paper-based tool. The tool order was randomized. Each session comprised 30 patient cards which had to be classified to one of the four triage categories. The 30 patient cards were distributed along a course of exactly 96.32m for each triage tool. The two sessions were conducted simultaneously, with the next participants starting their sessions every 5 minutes (see *Figure 2* for a sketch of the experimental setup, *Multimedia Appendix 1* for a drone picture and *Multimedia Appendix 2* for and drone film of the setting).
After each session, the participants completed a web-based questionnaire using the online questionnaire tool SurveyMonkey (SurveyMonkey Inc., San Mateo, California, USA), and then started the second session with the respective other triage tool after a recovery period of approximately 30 minutes.

Who provided: There were no direct "intervention providers" in our study, as the intervention was app-based and therefore delivered via individual mobile devices.

The introduction to both triage tools prior to the triage training was given by a well-trained paramedic instructor and emergency physician, and IT experts were present to answer questions about the app system or any technical problems that arose.

How (mode of delivery; individual or group): Participants downloaded and installed the KatApp on their mobile device prior to the study, and were introduced to the mobile-based system in a group. Afterwards, the participants underwent the two triage sessions individually, using either the KatApp or the paper-based tool.

Where: The study was conducted on the premises of the German Red Cross in Kirchentellinsfurt on behalf of the University Hospital Tübingen together with Reutlingen University in June 2024. The study participation was voluntary. Each Session consisted of 30 patient cards that were distributed along a course of 96.32m. Emergency vehicles and a fog machine blocked the view between the two courses. In addition, simulated sounds of a mass casualty incident were played via connected sound boxes that were placed in the middle of each course. Immediately after the sessions, the participants were asked to scan a QR code to complete the follow-up questionnaire.

When and how much: The study took place in June 2024 on a single day when participants were invited to a triage training course of the Tübingen District Association of the German Red Cross.

The study was conceived as a within-subjects experimental design, with the triage tool as within-subjects factor (paper-based tool vs. KatApp). The order of the triage tools was counterbalanced to compensate for carry-over effects.

The invitation to the triage training course and the call for participation in the study were sent to a mailing list of the Tübingen District Association of the German Red Cross which comprises approximately 500 different e-mail-addresses of rescue service personal, physicians and medical students with German Red Cross EMT qualifications.

After participants registered for the triage training course and study, they were provided with instructions on how to install the app and information regarding the study procedure one week prior to the course.

To familiarize the participants with the mSTART algorithm using both the paper-based tool as well as the KatApp, they were given an introduction at the beginning of the course (approx. 1 hour). They were then asked to complete two triage sessions in the context of a simulated mass casualty incident, which would be caused by a terrorist driving into the Fan Zone of a public screening of the UEFA European Football Championship 2024. In one session, they were using the KatApp, and in the respective other session the conventional paper-based tool. The tool order was randomized. Each session comprised 30 patient cards which had to be classified to one of the four triage categories. The 30 patient cards were distributed along a course of exactly 96.32m for each triage tool. The two sessions were conducted simultaneously, with the next participants starting their sessions every 5 minutes (see *Figure 2* for a sketch of the experimental setup, *Multimedia Appendix 1* for a drone picture and *Multimedia Appendix 2* for and drone film of the setting).

The participation in the study comprised 4 hours in total for each participant.

Tailoring: NA

Modification: NA

How well (planned): In total, 55 people initially registered for the course, while N=48 participants finally showed up and attended the course which displays a no-show rate of 12.7% (7/55). From those who attended the course, n=6 participants had to be excluded from further statistical analyses due to an incomplete documentation of the triages (n=3 with the paper-based tool, n=2 within the KatApp, n=1 within both tools), another n=2 participants did not complete questionnaire 1, and n=2 participants did not complete questionnaire 2. Hence, the final sample size consisted of N=38 participants for further statistical analyses.

How well (actual): In total, 55 people initially registered for the course, while N=48 participants finally showed up and attended the course which displays a no-show rate of 12.7% (7/55). From those who attended the course, n=6 participants had to be excluded from further statistical analyses due to an incomplete documentation of the triages (n=3 with the paper-based tool, n=2 within the KatApp, n=1 within both tools), another n=2 participants did not complete questionnaire 1, and n=2 participants did not complete questionnaire 2. Hence, the final sample size consisted of N=38 participants for further statistical analyses.
