# Supplementary material for: Evaluation of an App-Based Mobile Triage System for Mass Casualty Incidents: Within-Subjects Experimental Study
Source: J Med Internet Res. 2024 Nov 21;26:e65728. doi: 10.2196/65728 (PMC11621716; doi:10.2196/65728)
Supplement: Multimedia Appendix 9 [file jmir_v26i1e65728_app9.docx]

| Outcome variable | Paper-based, mean (SD) | KatApp, mean (SD) | *F* test (*df*) | *P* value | η^2^_p_ |
| --- | --- | --- | --- | --- | --- |
|  | Annotations | Annotations |  |  |  |
| Duration (minutes) | 43.71 (9.816) | 25.22 (4.737) | 229.769 (1, 36) | <.001 | 0.865 |
| Triage accuracy | 28.92 (1.099) | 29.45 (0.724) | 8.979 (1, 36) | .005 | 0.200 |
| Subjective rating | 3.26 (0.860) | 1.53 (0.506) | 116.281 (1, 36) | <.001 | 0.764 |
|  | Satisfactory^a^ | Excellent^a^ |  |  |  |
| **User Experience Questionnaire** |  |  |  |  |  |
| Attractiveness | –0.58 (1.175) | 1.96 (0.764) | 128.812 (1, 36) | <.001 | .782 |
|  | Bad^b^ | Excellent^b^ |  |  |  |
| Efficiency | –0.65 (1.224) | 1.86 (0.900) | 104.542 (1, 36) | <.001 | .744 |
|  | Bad^b^ | Good^b^ |  |  |  |
| Perspicuity | 0.94 (1.146) | 2.09 (0.861) | 26.390 (1, 36) | <.001 | .423 |
|  | Below average^b^ | Excellent^b^ |  |  |  |
| Dependability | 0.49 (0.937) | 1.88 (0.600) | 61.693 (1, 36) | <.001 | .631 |
|  | Bad^b^ | Excellent^b^ |  |  |  |
| Stimulation | 0.14 (1.251) | 1.76 (0.997) | 58.422 (1, 36) | <.001 | .619 |
|  | Bad^b^ | Excellent^b^ |  |  |  |
| Novelty | –1.45 (0.964) | 1.54 (0.999) | 132.687 (1, 36) | <.001 | .787 |
|  | Bad^b^ | Good^b^ |  |  |  |

^a^Grading according to the German academic grading system ranging from 1 (excellent) to 6 (insufficient).

^b^Classification according to the general benchmark of the User Experience Questionnaire [33].
